# Phoenixin-14 alters transcriptome and steroid profiles in female green-spotted puffer (*Dichotomyctere nigroviridis*)

**DOI:** 10.1038/s41598-022-13695-z

**Published:** 2022-06-08

**Authors:** Timothy S. Breton, Casey A. Murray, Sierra R. Huff, Anyssa M. Phaneuf, Bethany M. Tripp, Sarah J. Patuel, Christopher J. Martyniuk, Matthew A. DiMaggio

**Affiliations:** 1 Division of Natural Sciences, University of Maine at Farmington, Farmington, ME 04938 USA; 2 Tropical Aquaculture Laboratory, Program in Fisheries and Aquatic Sciences, School of Forest, Fisheries, and Geomatics Sciences, Institute of Food and Agricultural Sciences, University of Florida, Ruskin, FL 33570 USA; 3 Center for Environmental and Human Toxicology, Department of Physiological Sciences, College of Veterinary Medicine, University of Florida, Gainesville, FL 32611 USA

**Keywords:** Computational biology and bioinformatics, Molecular biology, Physiology, Endocrinology

## Abstract

Phoenixin (PNX) is a highly conserved, novel hormone with diverse functions, including hypothalamic control of reproduction, appetite modulation, and regulation of energy metabolism and inflammation. While some functions appear conserved across vertebrates, additional research is required to fully characterize these complex pleiotropic effects. For instance, very little is known about transcriptome level changes associated with PNX exposure, including responses in the hypothalamic–pituitary–gonadal (HPG) axis, which is critical in vertebrate reproduction. In addition, the PNX system may be especially complex in fish, where an additional receptor is likely present in some species. The purpose of this study was to assess hypothalamic and ovarian transcriptomes after PNX-14 administration in female vitellogenic green-spotted puffer (*Dichotomyctere nigroviridis*). Steroid-related changes were also assessed in the liver and blood plasma. Hypothalamic responses included pro-inflammatory signals such as interleukin 1β, possibly related to gut–brain axis functions, as well as suppression of cell proliferation. Ovarian responses were more widely downregulated across all identified pathways, which may reflect progression to a less transcriptionally active state in oocytes. Both organs shared regulation in transforming growth factor-β and extracellular matrix remodeling (periostin) pathways. Reproductive processes were in general downregulated, but both inhibiting (bone morphogenetic protein 15 and follistatin) and promoting (17-hydroxyprogesterone) factors for oocyte maturation were identified. Select genes involved in reproduction (vitellogenins, estrogen receptors) in the liver were unresponsive to PNX-14 and higher doses may be needed to induce reproductive effects in *D. nigroviridis*. These results reinforce the complexity of PNX actions in diverse tissues and highlight important roles for this hormone in regulating the immune response, energy metabolism, and cell growth.

## Introduction

Phoenixin (PNX) is a highly conserved, novel hormone first discovered using a bioinformatics approach to search the human genome for previously unknown secreted peptides^1^. PNX is a proteolytic cleavage product of small integral membrane protein 20 (SMIM20), also known as MITRAC7, which functions in respiratory chain assembly in mitochondria to chaperone cytochrome *c* oxidase^2–4^. The C-terminal domain of SMIM20 can be cleaved into several amidated forms, with the 14- and 20-amino acid peptides (PNX-14 and PNX-20, respectively) being the most predominant^4^. Both forms appear functionally similar and exhibit highest expression in the mammalian hypothalamus, with lower levels in the heart, thymus, and digestive system^1,5^. A putative receptor for PNX ligands was recently identified as GPR173, also known as SREB3, which is a member of the highly conserved G protein-coupled receptor family SREB (Super-conserved Receptors Expressed in Brain)^6–8^. PNX effects appear to require functional SREB3, although its status as the receptor is unclear since direct binding assays have been largely unsuccessful, and evolutionary studies may not support this relationship^7,9^. Overall, the PNX/SREB3 system has been associated with pleiotropic functions in numerous tissues, ranging from control of reproduction in the ovary and hypothalamus to stress and anxiety modulation, anti-inflammatory roles, appetite and thirst regulation, and glucose homeostasis^4,10^.

Some of the more well-studied functions of PNX are related to metabolism within the gut–brain axis. For example, intracerebroventricular PNX injection is associated with increased food intake in mice and rats and can lower core body temperature with anxiolytic effects^11–13^. Within stomach endocrine cells, PNX may stimulate ghrelin production and inhibit cholecystokinin, while pancreatic islet functions include insulin secretion and beta cell proliferation in rats^14,15^. Metabolic effects also extend beyond these sites, where PNX induces osteoblast differentiation in a mouse bone cell line and has anti-inflammatory properties in the small intestine^16,17^. Indeed, immune-related and proliferative roles may be critical in PNX/SREB3 signaling, since PNX promotes mitochondrial biogenesis and exhibits protective effects in the brain and heart^18–20^.

Despite recent research into PNX metabolic and immune functions, the hormone is likely most well known as a hypothalamic neuropeptide that promotes reproductive processes^1^. These functions include effects on the estrous cycle in rats, stimulation of gonadotropin releasing hormone (GnRH) and kisspeptin neurons in the hypothalamus, and luteinizing hormone (LH) release from the pituitary^1,7,8^. Overall, PNX seems broadly associated across the hypothalamic–pituitary–gonadal (HPG) axis and is also localized within the human ovary, inducing granulosa cell proliferation, estrogen production, and ovulation^21^. Some reproductive dysfunctions are also associated with the PNX/SREB3 system, including endometriosis, polycystic ovary syndrome in humans and rats, and cystic endometrial hyperplasia in dogs^22–25^.

The PNX/SREB3 system is far less-studied in non-mammalian vertebrates, but recent research has identified conserved roles in both appetite regulation and reproduction. For instance, hypothalamic PNX also affects food intake in chicken (*Gallus gallus*), spotted scat (*Scatophagus argus*), and zebrafish (*Danio rerio*)^26–28^. Many reproductive processes are also promoted by PNX in fish, including numerous gene expression increases in the hypothalamus, pituitary, ovary, and liver, as well as oocyte maturation in zebrafish^29,30^. However, data remains sparse for the role of PNX in vertebrates, especially in fish, where recent research identified a duplicated SREB3 gene (*sreb3b*) that is only found in some species^31^. The presence of a second SREB3 in PNX signaling may indicate the importance of this system in fish. Furthermore, to our knowledge, no study to date has assessed transcriptomic responses following PNX treatment in any vertebrate, which was recently identified as a research gap in our collective understanding of PNX signaling^10^. To this end, we determined the molecular effects of PNX-14 on the HPG axis in female green-spotted puffer (*Dichotomyctere nigroviridis*) using RNA-sequencing. PNX-14 was previously associated with reproductive changes in another perciform species^29^, and a dose was chosen (100 ng/g body weight) that is known to affect reproduction in two other fishes^29,30^. Puffer was chosen because it expresses both *sreb3* genes^31^ and is characterized by reproductive dysfunction in aquaculture conditions, where ovarian development progresses to vitellogenesis, but does not undergo oocyte maturation without artificial hormonal intervention^32^. Given its conserved role in the HPG axis, PNX may therefore be a useful hormone to regulate oocyte maturation in diverse fishes. We assessed hypothalamic and ovarian transcriptomes, along with steroid-related analyses in blood plasma and liver, to: (1) broadly characterize PNX-14 transcriptome effects, and (2) determine if treatment affected reproductive processes associated with oocyte maturation in vitellogenic pufferfish.

## Methods

### PNX-14 treatments and puffer sampling

Sexually mature female green-spotted puffers (8.6 ± 0.3 g, 60.3 ± 0.65 mm total length) of wild origin were imported through a local wholesaler (Segrest Farms, Inc.) and held in a freshwater flow through system at the University of Florida Tropical Aquaculture Laboratory (Ruskin, FL, USA). All puffers were maintained and sampled under approved guidelines of the University of Florida (UF) Institutional Animal Care and Use Committee (IACUC #: 202011293). All experimental protocols were performed in accordance with the University of Florida IACUC ethical guidelines and regulations for vertebrate animal use, as well as approval through the University of Maine at Farmington (UMF) Grant Coordination Committee. All experiments were conducted in compliance with ARRIVE guidelines.

To promote ovarian development to the vitellogenic stage, fish were slowly acclimated to saltwater (30 g/L salinity) over 3 months^32^. Fish (n = 12) were then anesthetized with 150 mg/L neutral-buffered tricaine methanesulfonate (MS-222), confirmed to be vitellogenic by ovarian biopsy^33^, and intramuscularly injected with puffer-specific PNX-14 (100 ng/g body weight, n = 6) or sterile water control (n = 6). PNX was diluted using sterile water, and the 100 ng/g body weight concentration was previously used in both spotted scat and zebrafish with significant HPG axis effects^29,30^. Briefly, to identify puffer-specific PNX, a spotted scat (*Scatophagus argus*) sequence (GenBank Acc. No. MH360732)^29^ was compared using BLAST to the puffer genome on Ensembl. The significant match (GSTENT10013530001) was confirmed manually to be identical and highly similar to *D. nigroviridis* and *Takifugu rubripes* sequences, respectively, in NCBI databases. The sequence was translated using ORFfinder (NCBI), and the PNX-14 peptide (DVQPVGMKIWSDPF-amide) was purchased and met all quality standards (≥ 98% purity) from Genscript USA, Inc. (Piscataway, NJ, USA).

Following injection, PNX-14 treated and control fish were held in individual 20 L containers housed within two 400 L culture tanks within a 1400 L recirculating system for 24 h, then euthanized with 200 mg/L neutral-buffered MS-222. All 20 L containers had small openings to allow for passive water flow and homogenous water quality parameters among replicates. To assess reproductive changes in response to PNX-14, ovarian biopsies and hypothalamic brain sections^34^ were preserved in RNALater (Ambion, Inc., Austin, TX, USA), snap frozen on dry ice, and stored at − 80 °C until subsequent RNA extractions at UF. To assess steroid-related patterns in response to PNX-14, the experiment was repeated using a second set of fish (n = 6 fish/treatment) and all individuals were euthanized 24 h following PNX-14 exposure. Liver samples were preserved in RNALater (Ambion, Inc., Austin, TX, USA), snap frozen on dry ice, and stored at − 80 °C until shipment to UMF for subsequent RNA extractions. Blood samples were collected in lithium heparinized vials following caudal ablation. Samples were centrifuged, and separated blood plasma (3–22 μl/fish) was frozen (− 20 °C) prior to steroid quantification at the UF Analytical Toxicology Core Laboratory.

### RNA preparation and RNA sequencing (RNA-seq)

Total RNA for each ovary and hypothalamus was isolated using TRIzol (Thermo Fisher Scientific, Waltham, MA USA) as per the manufacturer’s instruction for transcriptomics. Due to budget constraints, we did not sequence the liver transcriptome but turned to a focused qPCR approach for transcripts important for reproduction. RNA concentration was determined using the Qubit^®^ 2.0 Fluorometer (ThermoFisher/Invitrogen, Grand Island, NY, USA) and RNA quality was assessed using the Agilent 2100 Bioanalyzer (Agilent Technologies, Inc.). A total of 22 RNA samples were deemed high quality for RNA-seq library construction and consisted of six control hypothalami (HC group), six treatment hypothalami (HT), five control ovaries (OC), and five treatment ovaries (OT). Total RNA was used for mRNA isolation using the NEBNext Ploy(A) mRNA Magnetic Isolation module (New England Biolabs, catalog # E7490). RNA library construction was then performed with the NEBNext^®^ Ultra™ II Directional RNA Library Prep Kit for Illumina^®^ (New England Biolabs, catalog #E7760) according to the manufacturer's user guide. Individually prepared libraries were pooled by equimolar concentrations and sequenced by NovoSeq 6000 using 150 bp paired end reads (Illumina Inc., CA, USA). Sequencing was performed by Novogene Corporation (Beijing, China). Raw data (raw reads) of FASTQ format were first processed through fastp. In this step, clean data (clean reads) were obtained from raw data by removing reads containing adapter and poly-N sequences and reads with low quality. At the same time, Q20, Q30 and GC content of the clean data were determined (Supplementary Table S1). All downstream analyses were based on clean data with high quality.

The reference genome (TETRAODON 8.0) and gene model annotation files were downloaded from the Ensembl genome website browser directly. Paired-end clean reads were mapped to the reference genome using HISAT2 software. Feature counts were used to count the read numbers mapped to each gene, including known and novel genes. Reads Per Kilobase of transcript, per Million mapped reads (RPKM) of each gene was calculated based on the length of the gene and read count mapped (Supplementary Fig. S1). Ovary and hypothalamus transcriptomes were submitted to the NCBI Gene Expression Omnibus (GEO) database (GSE183029). DESeq2 was used for differential expression analysis between the OC and OT and between HC and HT groups. Differentially expressed gene lists with log2 fold changes and P-values are provided in the Supplementary Data. To provide the most complete gene name information for downstream pathway analyses, each differentially expressed gene list was further annotated manually. Briefly, both ovary and hypothalamus genes initially listed as unknown and differentially expressed across treatments (P < 0.05) were manually used in BLAST searches against the nucleotide (nt) database (NCBI) to identify significant matches to known genes in other fishes (e-value < 1e^−05^). If ten or greater matches in the database to other fish species were present, then each unknown gene was provided with a gene ID using Zebrafish Information Network (ZFIN) nomenclature. The revised ovary and hypothalamus differentially expressed gene lists (Supplementary Data) were then used in pathway analyses.

### Pathway analysis

Gene set enrichment analysis (GSEA) and subnetwork enrichment analysis (SNEA) were conducted in Pathway Studio 12.0 (Elsevier, Amsterdam, Netherlands). A total number of 12,766 ovary and 12,966 hypothalamus genes were successfully mapped to mammalian homologs using the official gene name. Gene set enrichment analysis proceeded with 1000 permutations to generate the distributions. Pathway Studio conducted statistical enrichment based upon ontologies and curated pathways. A two-sample nonparametric Kolmogorov–Smirnov test was used to compare the cumulative distributions of two data sets (networks) for differences. Subnetwork enrichment analysis or SNEA is designed around networks of common regulators of expression using known relationships (i.e., expression, binding, etc.) derived for experimental data and literature which are focused on gene hubs. A distribution of expression values is calculated by a permutation algorithm to obtain a “background” distribution followed by a statistical comparison between the sub-network data (query data) and the background distribution using a Mann–Whitney *U* test. For both GSEA and SNEA, a P-value is generated to indicate whether a process is statistically enriched in the query dataset relative to what is expected by random chance based upon the background distribution. Enrichment P-value for a gene seed was set at P < 0.05. GSEA and SNEA lists for both ovary and hypothalamus are provided in the Supplementary Data.

To broadly compare transcriptome responses across organs, gene sets common to both ovary and hypothalamus were identified, and highly significant sets were assessed by P < 0.01 and > 50% of total pathway entries measured in the dataset as significantly different. Highly significant sets were then manually assigned to broad functional categories, mostly based on information in the UniProt database (Ensembl, Hinxton, Cambridgeshire, UK). To further assess if organ transcriptional responses were associated with broad up- or downregulation, median fold changes of these gene sets were used in Chi-square tests with expected equal frequencies between the number of positive and negative median fold changes. To more fully characterize changes specific to reproductive processes, each organ dataset was also queried for significant gene differences associated with selected anatomical concepts or cell processes (e.g. ovarian follicle or reproduction, respectively). Lastly, for SNEA, highly significant subnetworks in both organs were characterized by > 60% of total neighbors measured in the dataset with P-value < 1e^−05^.

### Liver RNA extractions and cDNA synthesis

Liver RNA extractions were performed at UMF using Tri Reagent (Sigma-Aldrich, St. Louis, MO, USA) and standard phenol/chloroform procedures, followed by a third precipitation step using polyvinylpyrolidone (PVP) (2% PVP, 1.4 M NaCl) and 5 M LiCl to remove polysaccharide contamination^35^. Total RNA quantity and quality were assessed using a NanoDrop 1000 spectrophotometer (Thermo Fisher Scientific, Waltham, MA, USA) and 1.0% agarose gel electrophoresis. Total RNA (2.5 μg/sample) was treated with ezDNase and a 5 min incubation at 37 °C to remove genomic DNA contamination (Invitrogen, Carlsbad, CA, USA). In addition, ovary and hypothalamus total RNA previously extracted at UF were treated with TURBO™ DNase following manufacturer’s protocols (Thermo Fisher Scientific) and shipped frozen to UMF for later quantitative PCR (qPCR) confirmation of RNA-seq differential gene expression. Ovary and hypothalamus sample sizes were n = 3, 4, 5, and 6 for OC, OT, HC, and HT groups, respectively. Liver, ovary, and hypothalamus cDNA synthesis was conducted using the Superscript IV VILO kit (Invitrogen).

### Quantitative PCR (qPCR)

To confirm RNA-seq identified expression patterns, ovary and hypothalamus cDNAs were used in qPCR assays with a StepOne Plus Real Time PCR System and FAST SYBR™ Green Master Mix (Applied Biosystems, Waltham, MA, USA). Primers were designed using NCBI Primer-BLAST from genes of interest identified in the transcriptomes (Supplementary Table S2). A total of three hypothalamus genes (*dkk3a*, *dpydb*, and *tgfb3*) and seven ovary genes (*acta1*, *dpydb*, *isr2a*, *safb*, *slc31a1*, *slc26a2*, and *srl*) were assessed in qPCR. To assess steroid-related expression changes in the liver, six genes were assayed including estrogen receptors (*esr1*, *esr2a*, *esr2b*), sex hormone binding globulin (*shbg*), and vitellogenin genes (*vtgab* and *vtgc*), based on previous research in zebrafish^30^. Primers for liver genes (Supplementary Table S2) were designed similarly to above from sequences available in the *D. nigroviridis* genome (Ensembl) (ENSTNIT00000015435, ENSTNIT00000005784, ENSTNIT00000015555, ENSTNIT00000008218, ENSTNIT00000000661, and ENSTNIT00000003224, respectively). Primer sets were verified for intended product amplification using standard PCR and 2.0% agarose gel electrophoresis, following prior protocols^36^. Assays were normalized using *eef1a*, which did not exhibit significant differences in expression across treatments (P > 0.05). All qPCR assays consisted of 10 μl reaction volumes, 1.33 μl diluted template, 0.02–1.0 μM primers (depending on assay), and standard cycling conditions (95 °C for 10 min, 40 cycles of 95 °C for 15 s and 60 °C for 1 min). All samples were assayed in duplicate, and relative standard curves (run in triplicate) were made from pooled cDNA. Optimized linear standard curves consisted of four to six points, with approximately 90–100% PCR efficiency and single peak amplification in dissociation curve analysis. Standard qPCR negative controls (no template and no reverse transcriptase) were also used and exhibited no contamination (liver) or only negligible levels (most Ct > 30, and five or more cycles greater than most samples) in ovary/hypothalamus assays.

For all assays, qPCR results were expressed relative to the control (set to 1.0) and analyzed using relative quantification^37^. Data were expressed as mean ± standard error, and log-transformed relative expression levels were analyzed using two sample *t* tests in SYSTAT12 (Systat Software Inc., San Jose, CA, USA) and assessed at the P < 0.05 level. To identify correlations (P < 0.05) between RNA-seq normalized count data and relative qPCR expression, least squares linear regression analyses (SYSTAT12) were also conducted^38^.

### Plasma steroid quantifications

Blood plasma steroid levels were quantified using a liquid chromatography tandem mass spectrometry (LC–MS/MS) method at the UF Analytical Toxicology Core Laboratory previously validated for low volumes^39^. Briefly, a QTRAP 6500 linear ion quadrupole LC–MS/MS mass spectrometer (AB Sciex, Framingham, MA, USA) was used with a Shimadzu Scientific Instruments (Columbia, MD, USA) liquid chromatography (LC) system and a Kinetex© PS C18 column (2.6 μm particle size, 100 Å pore size, and 2.1 × 150 mm diameters). Injection volumes were 10 μl. Six steroids were quantified (progesterone, 17-hydroxyprogesterone, corticosterone, cortisol, testosterone, and 11-ketotestosterone). Estradiol (E2) was not measured due to low overall concentrations. Steroid concentrations (ng/μl plasma) were log-transformed, assessed for normality, and used in two-sample *t* tests in SYSTAT12 to detect significant differences (P < 0.05) between treatments.

## Results

### Ovary and hypothalamus transcriptomes

**Figure 1:**
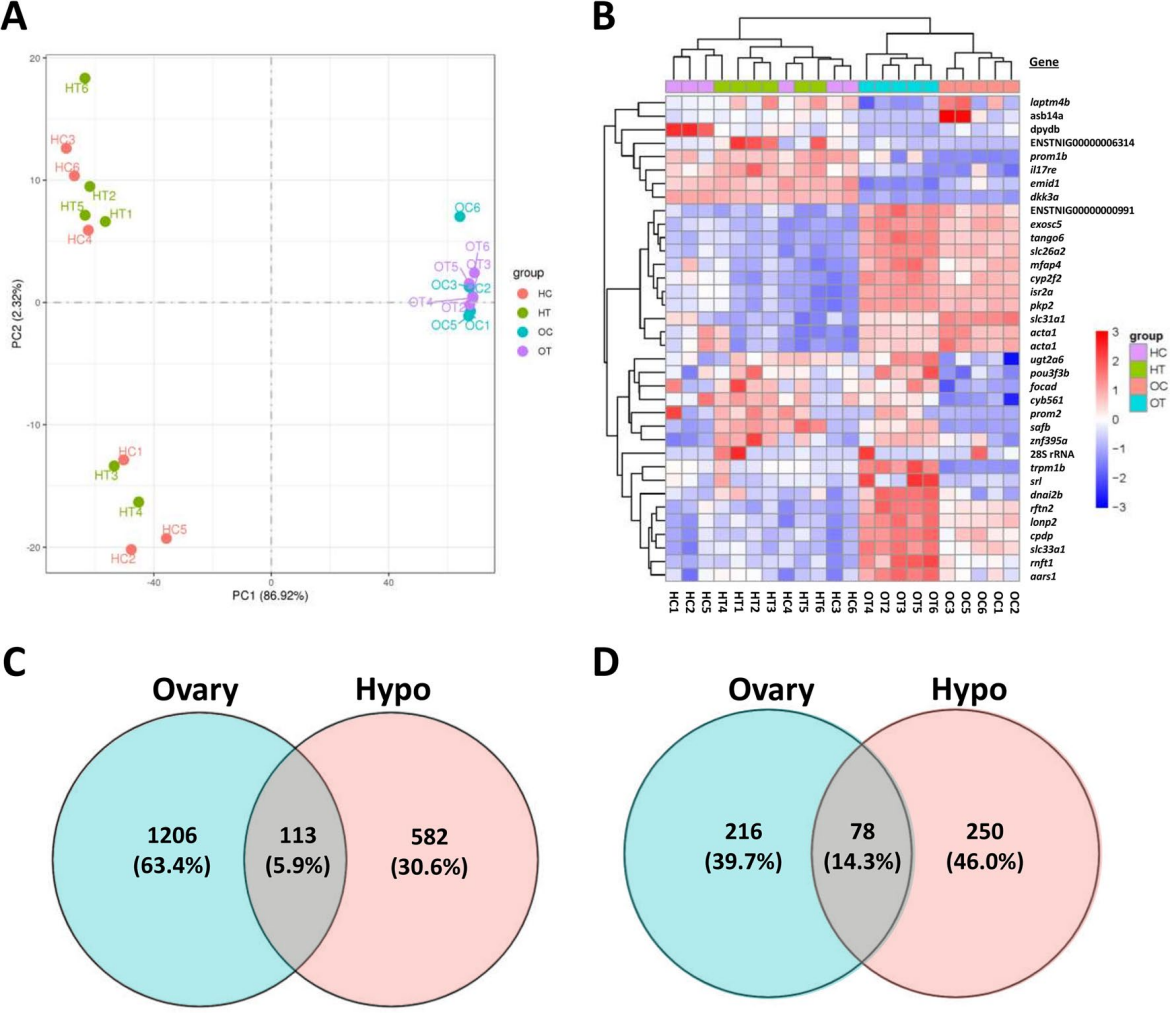
Principal component analysis (**A**) of transcriptomes in ovary control (OC, blue), ovary PNX-14 treated (OT, purple), hypothalamus control (HC, red) and hypothalamus PNX-14 treated (HT, green) samples. (**B**) Heat map of 36 differentially expressed genes (false discovery rate < 0.05) across both tissues, organized by genes (rows) and samples (columns). Gene names and individual sample IDs are provided on the right and bottom, respectively. Red colors indicate positive log2 fold changes in expression, while blue indicates negative changes. Unknown genes are labeled with their respective gene IDs identified in the Ensembl puffer genome (TETRAODON 8.0). (**C**) Total differentially expressed genes (P < 0.05) or (**D**) pathways with percentages (%) in the PNX-14 treatment in ovary (blue) and hypothalamus (red) transcriptomes, with shared entries across both transcriptomes identified in the middle (purple).

Ovary and hypothalamus transcriptomes were overall high quality, generating 20.2–27.0 million raw reads per sample that were processed to 19.8–26.5 million clean reads per sample (Supplementary Table S1). Error rates were low for all samples (0.03%) and Q20 and Q30 quality scores were high (mean 97.5% and 93.3%, respectively). Mapping rates to the puffer reference genome were also high (mean 90.0% and 85.5% total and uniquely mapping rates, respectively) and fragments per kilobase of transcript per million mapped reads (FPKM) levels were similar across samples (Supplementary Fig. S1). Mapping identified 20,562 total unique gene sequences across both organ types. Ovary and hypothalamus transcriptome profiles exhibited high divergence from each other but overall low discrimination between control and PNX-treated samples within an organ type (Fig. 1A). In addition, hypothalamus samples were separated into two groups that did not reflect treatments (HC and HT labels), although the variation along this component was far less (2.32%) than that between organs (86.92%). Differential gene expression analysis was used to identify 36 total genes with highly significant (P adj. < 0.05) up- or downregulation following PNX-14 treatment, which were divided into 31 ovary genes and five hypothalamus genes (Fig. 1B, see Supplementary Data for gene lists). Ovary samples exhibited clearer discrimination by treatment among highly significant genes (Fig. 1B, top blue and orange labels), while hypothalamus samples overall did not group by treatment. Among all differentially expressed genes (raw P-value < 0.05), the ovary and hypothalamus exhibited totals of 1206 and 582 genes, respectively, with 113 common genes between organ types (Fig. 1C). Following pathway analyses, these gene sets were categorized into 216 and 250 unique pathways with significant up- or downregulation (P < 0.05) in the ovary and hypothalamus, respectively (Fig. 1D). A total of 78 significantly different pathways were shared between organ types (Supplementary Data).

**Figure 2:**
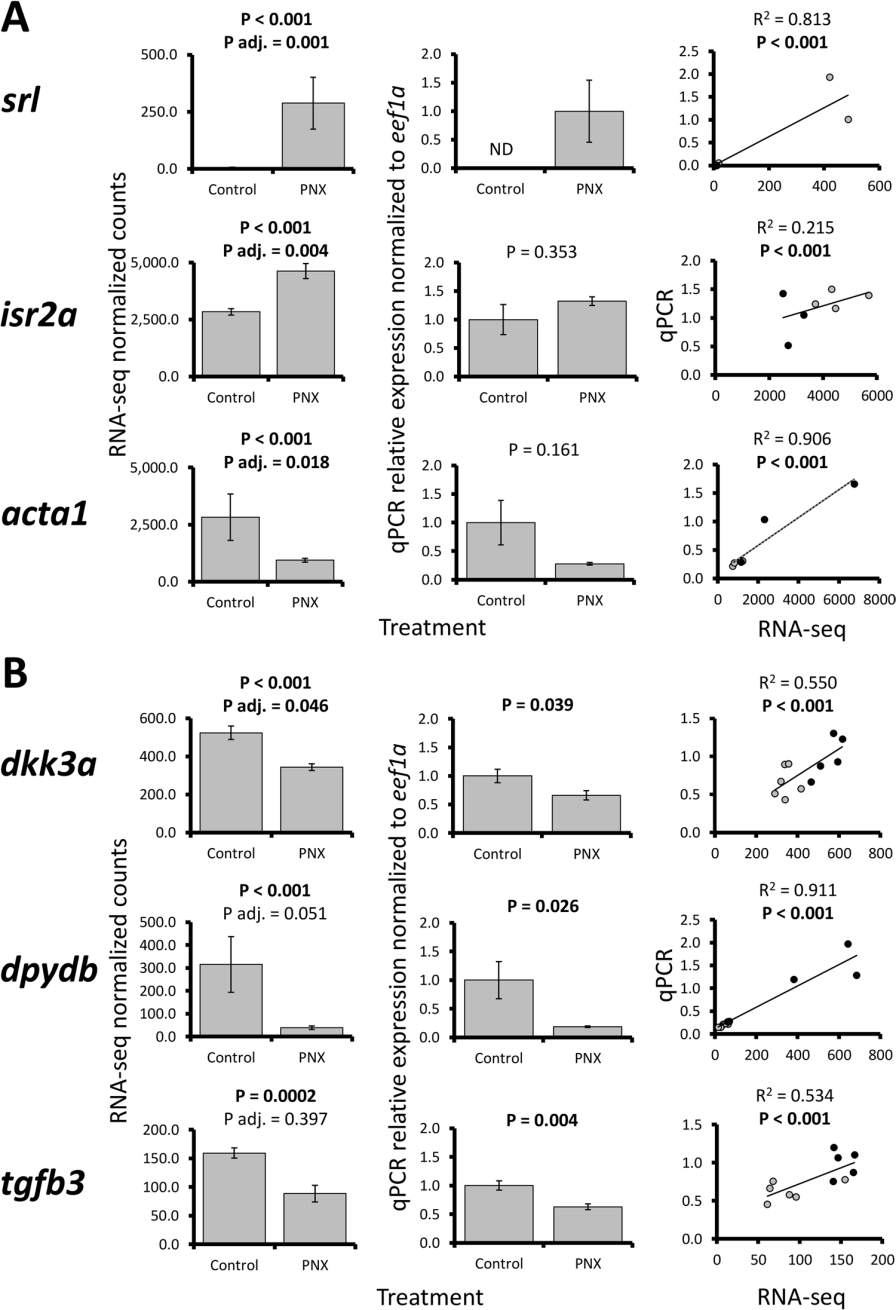
RNA-seq normalized count data (first column), relative mRNA expression in qPCR normalized to *eef1a* (second column), and linear regression analyses (third column) of selected ovary [(**A**) *srl*, *isr2a*, and *acta1*] and hypothalamus genes [(**B**) *dkk3a*, *dpydb*, and *tgfb3*]. Each bar represents the mean ± standard error and significance was assessed at P < 0.05. P adjusted (adj.) refers to the multiple test correction in RNA-seq differential expression analyses (false discovery rate < 0.05). For regression analyses, black and gray circles refer to control and PNX-14 treated samples, respectively.

Selected ovary and hypothalamus gene patterns were largely confirmed in qPCR (6 of 10 total assays, Fig. 2). Ovary patterns were variable, where three genes (*srl*, *isr2a*, and *acta1*) exhibited significant positive correlations between RNA-seq and qPCR data but did not have significant changes in relative expression (Fig. 2A). Four other ovary genes (*safb*, *slc31a1*, *slc26a2*, and *dpydb*) did not exhibit positive correlations (Supplementary Fig. S2), which were likely due to overall low sample sizes and fold changes for most genes (< 2.0). However, all three hypothalamus genes assayed (*dkk3a*, *dpydb*, and *tgfb3*) were confirmed across RNA-seq and qPCR techniques, with significant expression changes and positive correlations (Fig. 2B).

### Transcriptome networks

**Figure 3:**
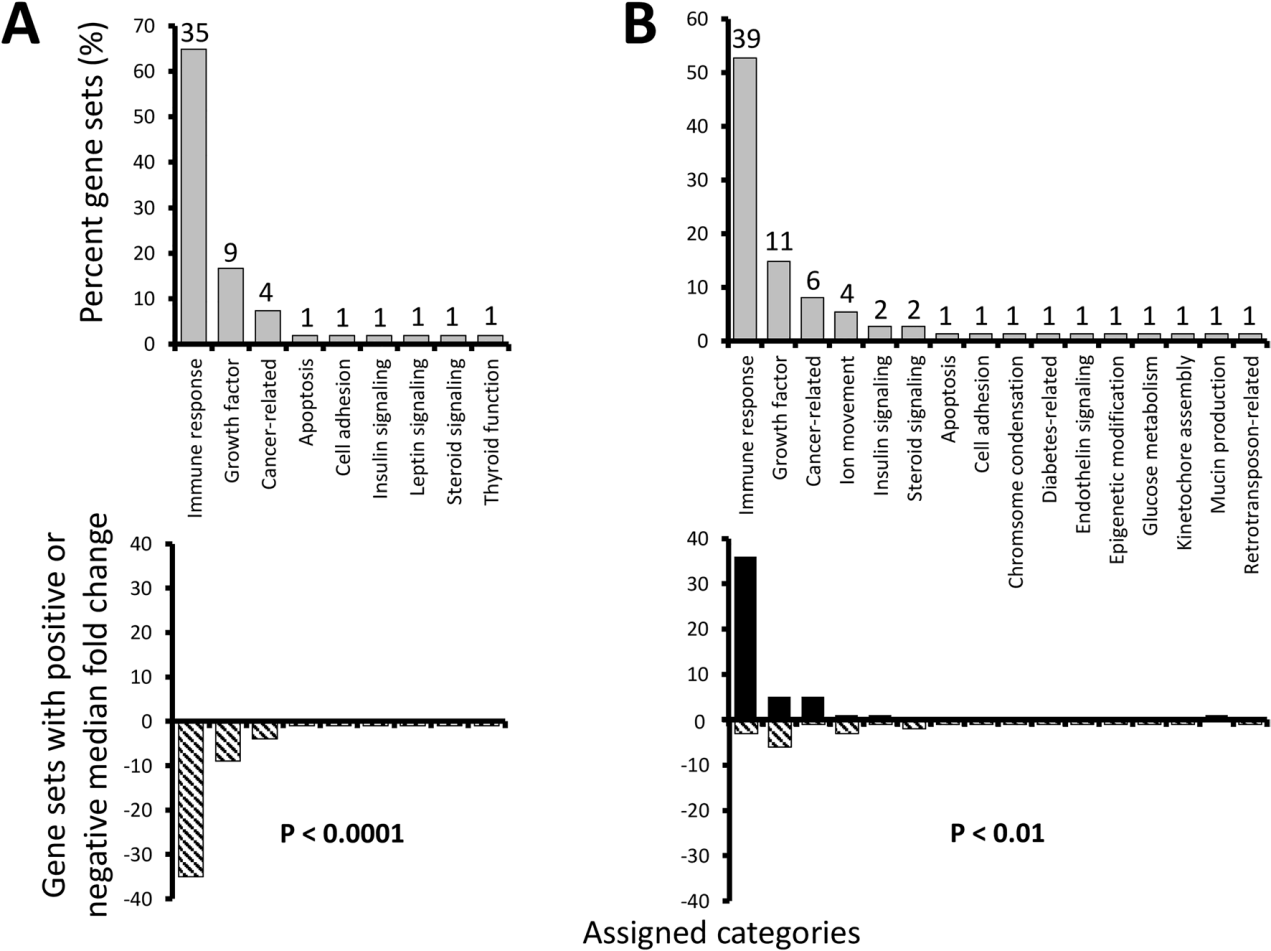
Percent gene sets (%) in the ovary (**A**) and hypothalamus (**B**) identified as highly significant (P < 0.01 with > 50% measured entries in a pathway) following PNX treatment were manually assigned functional categories (upper graphs). Numbers above gray bars refer to the total gene set number assigned to each category. Lower graphs identify the percent of gene sets (%) with overall positive (black bars) or negative (striped) median fold changes in each category. P values refer to significant differences from an expected equal frequency of gene sets with positive and negative changes.

Both ovary and hypothalamus exhibited changes in numerous gene pathways following PNX exposure, including highly significant shifts in 54 and 74 gene sets, respectively (Supplementary Data). When these were grouped into functional themes, both organs exhibited a majority of changes likely related to immune responses, including natural killer cell activation, T cell receptor pathways, and cytokine signaling. Overall, 35 and 39 highly significant gene sets were associated with immune responses in the ovary and hypothalamus, respectively (Fig. 3). The second and third most abundant categories were also similar in theme across organs, with nine and 11 sets associated with growth factors (e.g. transforming growth factor-α and amphiregulin pathways), and four and six sets being cancer-related (e.g. “ERBB/VEGFR/Akt signaling in breast cancer” and “Hodgkin lymphoma”). Less abundant categories varied more between organs, but some were still shared such as insulin signaling, steroid signaling, apoptosis, and cell adhesion, even though the specific gene sets within those themes often varied. Other categories related to leptin signaling and thyroid function were unique to the ovary, while ion movement, chromosome condensation, diabetes-related, endothelin signaling, epigenetic modification, glucose metabolism, kinetochore assembly, mucin production, and retrotransposon-related were unique to the hypothalamus. Overall, even though some categories were shared between organs, the PNX response differed greatly. The ovary was characterized by entirely negative median fold changes in highly significant gene sets (P < 0.0001), while the hypothalamus exhibited mostly increases (P < 0.01), especially in immune response (Fig. 3).

**Figure 4:**
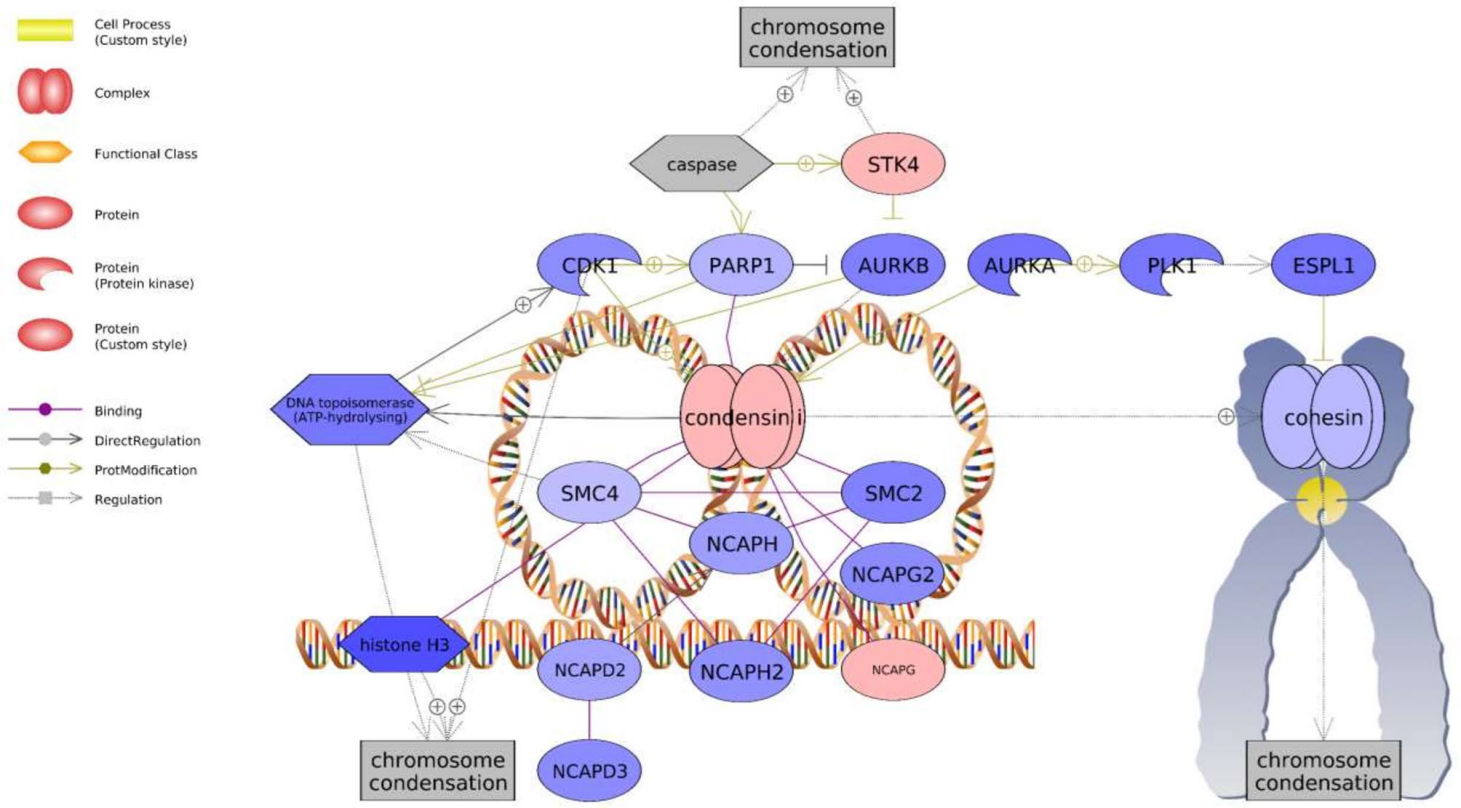
Chromosome condensation-associated network identified in the hypothalamus following PNX treatment. Red indicates an upregulated gene in the network (darker shade indicates greater log2 fold change compared to control), while blue indicates a downregulated gene. Shapes and arrows are identified in the legend to the left. This is a proposed mechanism based only on transcriptomic data.

Many highly significant gene sets were associated with known PNX roles in energy metabolism, nociception, reproduction, and inflammatory response (Table 1). These sets included “LIF Expression Targets” (− 1.16 median fold change) and “InsulinR → CTNNB/FOXA/FOXO Signaling” (− 1.14) in the ovary, and “IL1B Expression Targets → Nociception” (1.07) and “Estrogens in Prolactin Production” (− 1.05) in the hypothalamus. In particular, the “Chromosome Condensation” gene set was among the greatest hypothalamic median fold changes detected (− 1.22) and also exhibited 100% representation, with 21 genes exhibiting changes out of 21 total genes in the pathway (Table 1). Overall, most genes in the chromosome condensation network exhibited expression decreases, except for increases in serine-threonine kinase 4 (*stk4*) and non-SMC condensin I complex subunit G (*ncapg*) (Fig. 4).

**Table 1:** Selected highly significant gene sets (P < 0.01 with > 50% measured entities in a pathway) following PNX-14 treatment. Five selected gene sets unique to ovary and hypothalamus are provided, followed by six total sets shared between organs. See Supplementary Data for full lists. Total entities, measured entities, and percent (%) refer to the total number of genes in the set, number of those genes detected with significant fold expression changes, and the percentage, respectively. Median change refers to the median positive or negative log2 fold change across all measured entities in that gene set. For shared gene sets, ovary and hypothalamus values are provided before and after slashes, respectively.

Name	Total entities	Measured entities	Percent (%)	Median change	P-value
**Selected ovary gene sets**					
TGFA → CTNNB/CTNND Expression Targets	36	21	58.3	− 1.06	0.0020
LIF Expression Targets	43	22	51.2	− 1.16	0.0019
InsulinR → CTNNB/FOXA/FOXO Signaling	14	9	64.3	− 1.14	0.0056
IGF1R/AKT Signaling in Breast Cancer	49	44	90.0	− 1.14	0.0089
Androgen Receptor/SGK1 Signaling	17	10	58.8	− 1.28	0.0094
**Selected hypothalamus gene sets**					
AREG → FOXO3A Expression Target	11	7	63.6	− 1.02	0.0018
Chromosome Condensation	21	21	100.0	− 1.22	0.0019
IL1B Expression Targets → Nociception	56	29	51.8	1.07	0.0022
NF-kB Canonical Signaling	62	51	82.2	1.11	0.0024
Estrogens in Prolactin Production	29	23	79.3	− 1.05	0.0058
**Total shared highly significant gene sets**					
Gamma Globulins Expression Targets	48	46/45	95.8/93.8	− 1.16/1.03	0.0096/0.0046
IL1B Expression Targets	169	95/93	56.2/55.0	− 1.14/1.07	0.0040/0.0069
Natural Killer Cell Activation through ITAM-Containing Receptors	86	60/57	69.8/66.3	− 1.15/1.05	0.0018/0.0045
Periostin (POSTN) Production by Airway Epithelium in Asthma	17	13/13	76.5/76.5	− 1.44/− 1.07	0.0080/0.0076
TCR → STAT Expression Targets	41	24/24	58.5/58.5	− 1.16/1.11	0.0078/0.0045
TGFB1-TGFBR1/AP-1 Expression Targets	123	87/86	70.7/69.9	− 1.25/1.06	0.0041/0.0089

**Figure 5:**
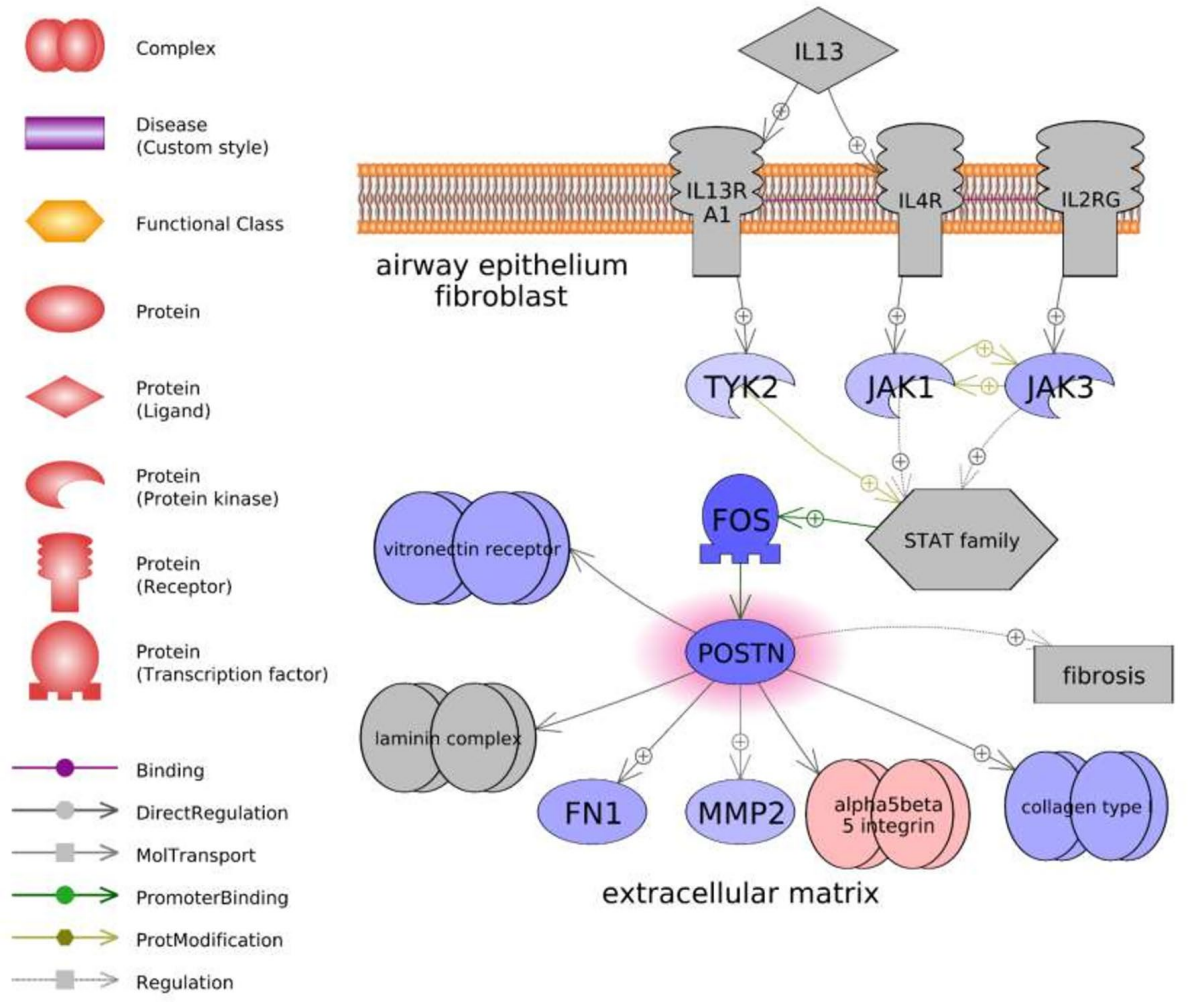
Periostin (*postn*)-associated network identified in the ovary following PNX treatment. Red indicates an upregulated gene in the network (darker shade indicates greater log2 fold change compared to control), while blue indicates a downregulated gene. Shapes and arrows are identified in the legend to the left. This is a proposed mechanism based only on transcriptomic data.

Of all highly significant gene sets, only six were shared in both organs (Table 1). These included four likely immune response pathways (“gamma Globulins Expression Targets,” “IL1B Expression Targets,” “Natural Killer Cell Activation through ITAM-Containing Receptors,” and “TCR → STAT Expression Targets”) one growth factor-associated pathway (“TGFB1-TGFBR1/AP-1 Expression Targets”), and one cell adhesion pathway (“Periostin (POSTN) Production by Airway Epithelium in Asthma”). Five of these sets exhibited differences in median fold changes between organs, with the immune- and growth-related pathways more greatly decreasing in the ovary and slightly increasing in the hypothalamus. For example, the transforming growth factor-β (*tgfb*) associated network was characterized by more extensive downregulation in the ovary, with upstream decreases in both the *tgfb1* ligand and a receptor (*tgfbr2*) (Supplementary Fig. S3). In contrast, the same network in the hypothalamus was characterized by increases in these genes, along with broad increases in many downstream targets (Supplementary Fig. S4). Only the *postn*-associated pathway exhibited similar changes across both organs (− 1.44 vs. − 1.07 downregulation) and was characterized by 76.5% of the total genes changing in response to treatment. The changes in this network were associated with decreases in tyrosine-protein kinases (*tyk2*, *jak1*, and *jak3*), a gene with similarity to proto-oncogene c-Fos (*fos*), periostin (*postn*), and several downstream targets (Fig. 5).

Both the ovary and hypothalamus also exhibited evidence of downregulation in reproductive processes. When the ovary transcriptomes were queried for genes associated with anatomical concepts in reproduction, a network was identified with 85 genes that were largely downregulated in response to PNX-14 (Supplementary Fig. S5). Of note, this network included increases in hormones associated with reproductive processes in the ovary (e.g. bone morphogenetic protein 15, *bmp15*; gonadotropin-releasing hormone 2, *gnrh2*) but decreases in many transcription factor genes. There were no changes in steroidogenic enzymes, except for an increase in 17-beta-hydroxysteroid dehydrogenase 7 (*hsd17b7*). In contrast, a smaller reproductive network (17 genes) was identified in the hypothalamus and was based on cell processes of reproduction, lactation, and apoptosis (Supplementary Fig. S6). This network was also largely downregulated and included decreases in a dopamine receptor (*drd2*), estrogen receptors (*esr1* and *esr2*), prolactin (*prl*), and luteinizing hormone. Some growth factors associated with reproductive processes varied, such as increases in *tgfb1* but decreases in transforming growth factor-β 3 (*tgbf3*) and fibroblast growth factor 2 (*fgf2*).

Regarding subnetworks, a total of 50 were both highly significant and shared across ovary and hypothalamus (see SNEA in Supplementary Data), with a smaller set associated with reproduction. These subnetworks were also downregulated and included regulators of hormone secretion (− 1.09 and − 1.02 median change in ovary and hypothalamus, respectively), fertilization (− 1.05 and − 1.02), and ovary follicle maturation (− 1.15 and − 1.00). Many other subnetworks were also shared between organs, with some of the most highly significant in both being regulators of chemotaxis (− 1.09 and 1.02) and cell interaction (− 1.10 and − 1.00). In addition, two subnetworks related to regulators of pregnancy (− 1.07 and − 1.01) and calcium mobilization (− 1.08 and 1.00) also exhibited high P-values in both organs (> 1e^−08^) but were less represented among measured neighbors (57–59%). Overall, other top subnetworks ranked by P-value in the ovary focused on decreases in neural signals (e.g. excitability, transmission of nerve impulse, and perception of pain) while top subnetworks in the hypothalamus were increases related to immune response (e.g. leukocyte activation, NK cell mediated cytotoxicity, and immune system function).

### Steroid-related patterns in liver and blood plasma

**Figure 6:**
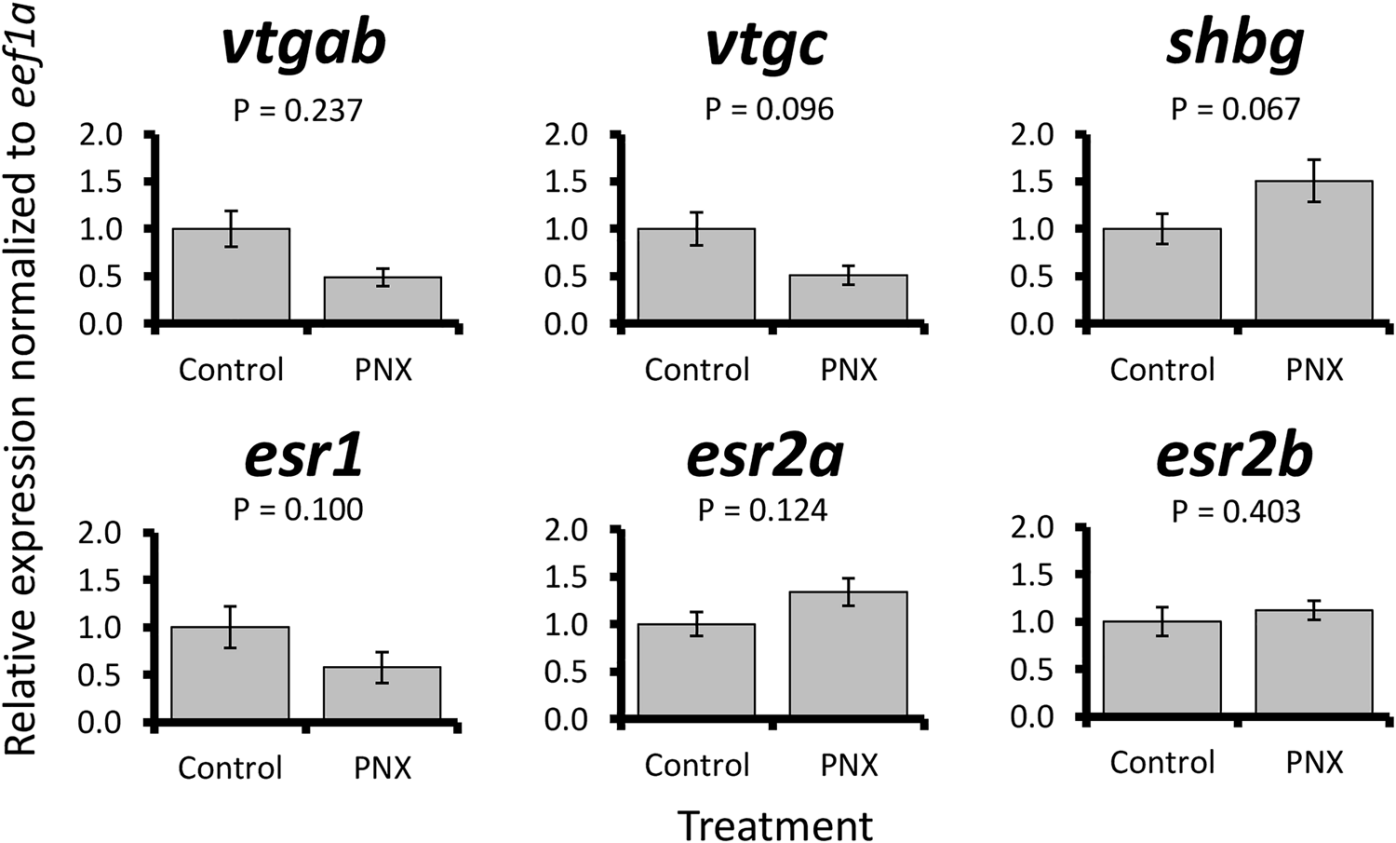
Relative mRNA expression normalized to *eef1a* of six steroid-associated liver genes (*vtgab*, *vtgc*, *shbg, esr1*, *esr2a*, and *esr2b*) in control and PNX-treated puffers. Each bar represents the mean ± standard error, and significance was assessed at P < 0.05.

**Figure 7:**
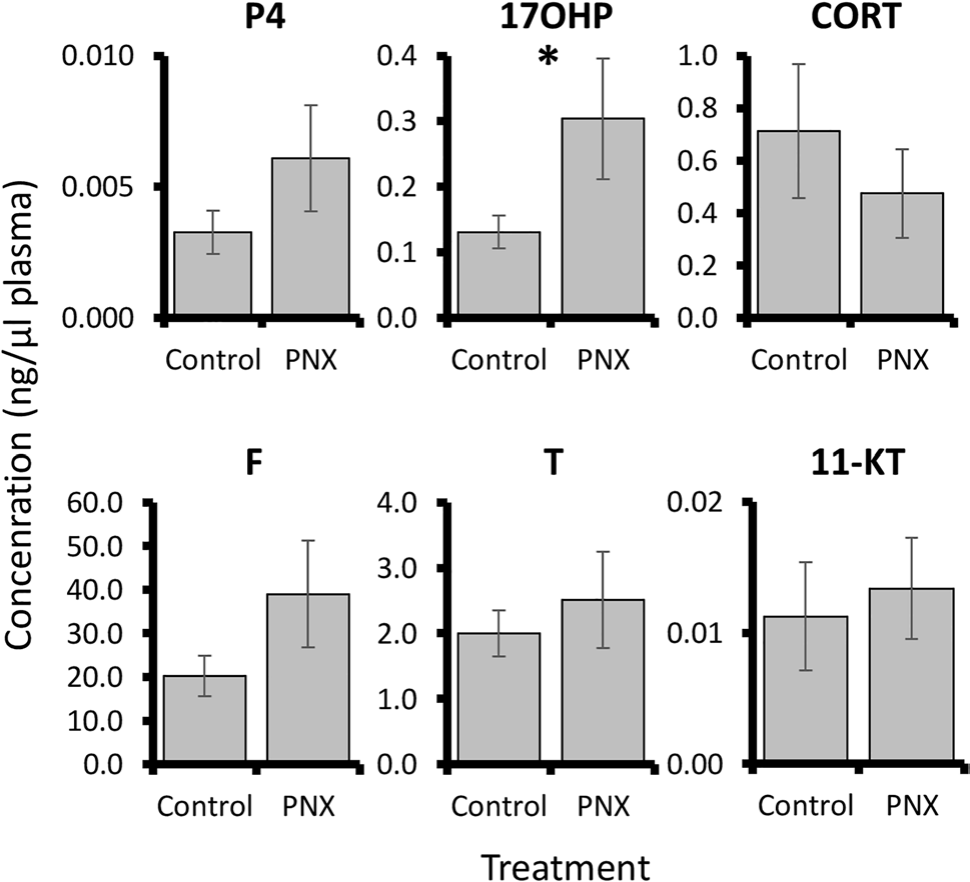
Blood plasma concentrations (ng/μl plasma) of six steroids (P4, progesterone; 17OHP, 17-hydroxyprogesterone; CORT, corticosterone; F, cortisol; T, testosterone; 11-KT, 11-ketotestosterone). Each bar represents the mean ± standard error and the asterisk indicates significance (P = 0.049).

PNX-14 treatment was overall associated with small changes in steroid-related patterns. In the liver, no significant expression changes were detected in vitellogenin genes or estrogen receptors (Fig. 6). However, a marginal but non-significant increase (< 2-fold) in *shbg* was detected following treatment (P = 0.067). Blood plasma steroid concentrations were overall low for progesterone, corticosterone, cortisol, testosterone, and 11-ketotestosterone, but a significant threefold increase in 17-hydroxyprogesterone was detected in PNX-14 treated fish (P = 0.049, Fig. 7).

## Discussion

PNX-14 exhibited pleiotropic actions in puffer, ranging from immune regulation to effects on cell growth, glucose metabolism, and reproductive processes. In particular, the most dramatic response in both ovary and hypothalamus was related to immunity, although broad changes differed between organs and were largely anti- or pro-inflammatory, respectively. PNX roles as an anti-inflammatory peptide are just beginning to be understood, including suppressive effects on cytokines and inflammation mediators in a variety of cell types^19,40–43^. Across these studies, many observed effects were similar to ovary changes in puffer, such as decreases in interleukin-1β, interleukin-6, toll-like receptor 4, and nuclear factor kappa B-associated pathways. The suppressive effects of PNX on inflammation processes may be highly conserved across vertebrates; however, this is also likely context dependent, since these same pathways were also detected within the hypothalamus, but were upregulated. The pro-inflammatory hypothalamic signal may instead be associated with energy metabolism and the gut–brain axis. For instance, hypothalamic inflammation is known to affect energy balance and contribute to insulin resistance^44,45^. Indeed, administration of another gut-associated peptide (glucose-dependent insulinotropic peptide, GIP) can both increase interleukin-1β and interleukin-6 in the mouse hypothalamus and impair insulin effects^45^. The putative PNX receptor, SREB3, has also been implicated in inflammatory roles within the mammalian brain, where the receptor is both critical to phoenixin effects in both astrocytes and microglia, and can be induced by an interleukin^19,46^. Spatially, SREB3 expression has been confirmed within the supraoptic and paraventricular nuclei of the hypothalamus in several mammals^47^, while PNX patterns are also present in the hypothalamus and strongly conserved across mammals and zebrafish^48^. Although more research is required to better understand these processes, especially in puffers, the identification of these same pathways possibly reinforce a hypothalamic link between immune response and energy homeostasis.

PNX-14 effects on cell growth, metabolism, and energy homeostasis were also evident. For instance, both ovary and hypothalamus exhibited changes in insulin-associated pathways, as well as transforming growth factor β (TGFβ), which is a cytokine with well-established roles in immune cell production and broad suppression of cell proliferation^49^. Indeed, TGFβ may be highly conserved as part of the PNX/SREB3 response, as SREB3 also decreases TGFβ signaling in mouse neurons and promotes cell migration^50^. Cell adhesion and extracellular matrix remodeling are likely part of the PNX response, since a periostin (POSTN)-associated pathway was the only highly significant gene set shared between organs and similarly downregulated. Periostin is an extracellular matrix protein that interacts with integrins to support cell adhesion and spreading, functioning to accelerate cell growth and oppose apoptosis^51^. Periostin is particularly prevalent in bone, teeth, and heart, where it promotes cell differentiation and repair, which may serve as one downstream mediator of the beneficial PNX effects in these tissues^16,18,41,51^. In puffer hypothalamus, decreases in the periostin pathway along with increases in TGFβ1 indicate an overall anti-proliferative signal. This is also supported by the dramatic downregulation of the chromosome condensation pathway, which would restrict mitotic spindle assembly and completion of mitosis^52^. Coupled with downregulation of other proliferation pathways, such as that associated with amphiregulin (AREG)^53^, the hypothalamic response to PNX-14 was largely focused on greater inflammation while down regulating some cell growth and metabolic processes.

PNX/SREB3 system roles in regulating cell growth and energy metabolism may be conserved among the entire SREB family of receptors^6^. Although other SREBs remain orphan receptors with no known endogenous ligands, they have independently been associated with similar processes. For instance, SREB1 (orphan receptor GPR27) is also known to affect insulin regulation and glucose tolerance^54,55^. SREB2 (orphan receptor GPR85) is a negative regulator of brain size, and SREB2 overexpression in mice reduces dendritic growth and neuronal connections^56,57^. In addition, teleost fish SREBs exhibit largely similar expression patterns across organs. For instance, patterns for the potential PNX receptor *sreb3a* in puffer closely match *sreb1*, with greatest expression in both brain and gonads^31^. The puffer brain also exhibits particularly elevated levels of *sreb3b*, a potential second PNX receptor, and these patterns are similar to *sreb2*^31^. In this study, the most significantly enriched ovary subnetworks were associated with neuron function, including neuronal plasticity, which is most closely associated with SREB2 functions^56^. As such, these receptor family responses may be more similar than currently studied and will require more investigation.

Although inflammation and growth regulation is important in PNX signaling, the ovary was more broadly characterized by near universal downregulation, with almost all identified pathways and subnetworks decreasing, irrespective of functional category. This may reflect a stage specific response, since puffer ovaries contained high proportions of mid or late vitellogenic follicles. Late oocyte growth stages in mice are much less transcriptionally active than earlier stages, and this is also observed in some fish^58,59^. In addition, PNX-exposed vitellogenic ovaries in zebrafish exhibited relatively fewer expression changes than other organs^30^. Overall, this supports the hypothesis that the cellular responses to PNX are context and stage dependent within a tissue. For example, unlike in the hypothalamus, the chromosome condensation pathway was not affected in the puffer ovary, where it is likely essential for oocyte meiotic progression and may be inversely related to transcriptional activity^58^. In addition, puffer ovarian responses to PNX may have differed greatly if earlier stages were used, which is supported by prior research in mammalian follicles where PNX promotes early follicular growth^21^.

Reproductive processes in both ovary and hypothalamus were largely downregulated in response to PNX, which differs from prior work in many vertebrates. For example, both hypothalamic neurons and ovarian follicle cells exposed to PNX upregulate expression patterns associated with the HPG axis in both mammals and fish^1,21,30^. In zebrafish, similar concentrations of PNX-20 to those used in the present study induced expression changes in reproductive hormones, receptors, and steroidogenic enzymes, although higher concentrations produced greater responses in some cases^30^. It is possible that the PNX-14 concentration used here may have been too low to exert strong reproductive effects in puffer, which is supported by a lack of hypothalamic changes in spotted scat at an identical concentration^29^. Liver responses may also support this, where puffer vitellogenin and estrogen receptor genes were unchanged, and zebrafish cell cultures only exhibited dramatic changes at very high media concentrations^30^. Still, some reproduction-associated pathways and subnetworks in both ovary and hypothalamus were altered by the PNX-14 treatment, including several associated with oocyte maturation.

Oocyte maturation in fish involves complex interactions across the HPG axis, including increases in pituitary luteinizing hormone (LH) release, a steroidogenic shift in gonad somatic cells, and production of other regulatory factors within the follicle^60,61^. For instance, elevated BMP-15 prevents precocious maturation, while the activin system and transforming growth factor alpha (TGFα) promote it^61,62^. In the present study, many of these associated genes or pathways were identified but overall indicate a suppressive effect, including increases in ovary *bmp15* and follistatin (*fst*), an inhibitor of activin, in addition to an overall decrease in a TGFα pathway. In addition, leukemia inhibitory factor (LIF) promotes oocyte maturation in some mammals^63^, but an LIF-associated pathway was strongly downregulated in the puffer ovary. Steroidogenic enzymes were also largely unaffected by the treatment, and subnetworks indicated a significant decrease in fertilization and oocyte maturation processes. In addition, hypothalamic changes indicated a possible decrease in overall LH expression. However, not all processes were suppressive, as PNX did induce elevated blood plasma levels of 17-hydroxyprogesterone, which is a precursor to maturation-promoting progestogens in fish^60^. Collectively, PNX appears to regulate oocyte maturation in puffer, but this may differ from other species^21,30^. These contrasting roles may be due to the duplicated receptor (SREB3B) in puffer, which is not found in zebrafish and mammals, or possibly due to culture conditions that limit maturation capacity. In either case, functional studies in puffer ovaries and higher PNX-14 doses are warranted to more fully decipher the roles of this novel hormone in oocyte maturation across vertebrates.

Overall, this study represents a foundational step in our broader understanding of PNX functions, and more research is needed across vertebrates to comprehensively characterize these conserved roles. For instance, the transcriptomic approach was conducted after 24 h, and there are likely early and late expression waves resulting in secondary or tertiary effects downstream of the transcriptome. We hypothesize that PNX regulates the inflammatory response, growth factors, and steroid signals initially, but additional experiments are required to elucidate temporal responses due to PNX-14. Such modulators likely have action sites beyond the hypothalamus and ovary, and possibly include changes within the gut or pituitary that have not yet been studied in this species. Further work is also required to better understand the increase in plasma 17-hydroxyprogesterone, which may have resulted from direct ovary changes related to oocyte maturation or indirect effects through the hypothalamus and pituitary. As such, in vitro approaches using ovarian fragments may provide additional insights into local responses. Dose responses may also vary both within a species and across vertebrates, and this warrants careful consideration in future comparative research where PNX effects may be contradictory^10^. This is especially relevant within the context of the present study, where higher doses or alternative forms of phoenixin (PNX-20) should be evaluated in puffer to assess changes in reproductive or immune effects. Lastly, more foundational work is still needed to better understand this system in fish, including investigations into PNX/SREB3 spatial patterns within brain regions, which have only recently been evaluated in zebrafish and may exhibit differences across fish evolution^31,48^.

## Conclusion

PNX-14 treatment in puffers induced broad changes related to immune responses, cell growth, and reproduction in both ovary and hypothalamus. Hypothalamic changes included a pro-inflammatory signal, possibly related to the gut–brain axis, and largely suppressive effects on proliferation. Ovary changes were characterized by broad decreases in numerous pathways that may reflect vitellogenic oocyte progression to a more transcriptionally inactive state. Both organs shared some pathways that may indicate conserved PNX roles, including regulation of the extracellular matrix and TGFβ signaling. PNX also downregulated some reproductive processes, and evidence for both inhibiting and promoting factors associated with oocyte maturation were identified. Further research in fish is warranted to thoroughly characterize the pleiotropic effects of this novel hormone, including investigations into differing dose responses and both in vivo and in vitro transcriptome and steroid changes.

## Supplementary Information

Supplementary Information 1.

Supplementary Information 2.

## Data Availability

The datasets generated and/or analyzed during the current study are available in the NCBI Gene Expression Omnibus (GEO) database, GSE183029.

## References

[1] Yosten GLC (2013). A novel reproductive peptide, phoenixin. J. Neuroendocrinol..

[2] Dennerlein S (2015). MITRAC7 acts as a COX1-specific chaperone and reveals a checkpoint during cytochrome *c* oxidase assembly. Cell Rep..

[3] McIlwraith EK, Belsham DD (2018). Phoenixin: Uncovering its receptor, signaling and functions. Acta Pharmacol. Sin..

[4] Billert M, Rak A, Nowak KW, Skrzypski M (2020). Phoenixin: More than a reproductive peptide. Intl. J. Mol. Sci..

[5] Prinz P (2017). Central and peripheral expression sites of phoenixin-14 immunoreactivity in rats. Biochem. Biophys. Res. Commun..

[6] Matsumoto M (2000). An evolutionarily conserved G-protein coupled receptor family, SREB, expressed in the central nervous system. Biochem. Biophys. Res. Commun..

[7] Stein LM (2016). Hypothalamic action of phoenixin to control reproductive hormone secretion in females: Importance of the orphan G protein-coupled receptor *Gpr173*. Am. J. Physiol. Reg..

[8] Treen AK, Luo V, Belsham DD (2016). Phoenixin activates immortalized GnRH and kisspeptin neurons through the novel receptor GPR173. Mol. Endocrinol..

[9] Yañez-Guerra LA, Thiel D, Jékely G (2022). Pre-metazoan origin of neuropeptide signaling. Mol. Biol. Evol..

[10] McIlwraith EK, Zhang N, Belsham DD (2022). The regulation of phoenixin: A fascinating multidimensional peptide. J. Endocr. Soc..

[11] Schalla M (2017). Phoenixin-14 injected intracerebroventricularly but not intraperitoneally stimulates food intake in rats. Peptides.

[12] Jiang JH (2015). Effects of pheonixin-14 on anxiolytic-like behavior in mice. Behav. Brain Res..

[13] Friedrich T (2019). Intracerebroventricular injection of phoenixin alters feeding behavior and activates nesfatin-1 immunoreactive neurons in rats. Brain Res..

[14] Billert M, Kołodziejski PA, Strowski MZ, Nowak KW, Skrzypski M (2019). Phoenixin-14 stimulates proliferation and insulin secretion in insulin producing INS-1E cells. BBA Mol. Cell Res..

[15] Murkherjee K, Unniappan S (2021). Mouse gastric mucosal endocrine cells are sources and sites of action of Phoenixin-20. Peptides.

[16] Gu Z, Xie D, Ding R, Huang C, Qiu Y (2020). GPR173 agonist phoenixin 20 promotes osteoblastic differentiation of MC3T3-E1 cells. Aging.

[17] Zandeh-Rahimi Y, Panahi N, Hesaraki S, Shirazi-Beheshtiha SH (2022). Protective effects of phoenixin-14 peptide in the indomethacin-induced duodenal ulcer: An experimental study. Int. J. Pept. Res. Ther..

[18] Rocca C (2018). Pheonixin-14: Detection and novel physiological implications in cardiac modulation and cardioprotection. Cell. Mol. Life Sci..

[19] Wang J (2020). The protective effects of phoenixin-14 against lipopolysaccharide-induced inflammation and inflammasome activation in astrocytes. Inflamm. Res..

[20] Yang Y (2020). Phoenixin 20 promotes neuronal mitochondrial biogenesis via CREB–PGC-1α pathway. J. Mol. Histol..

[21] Nguyen XP (2019). Effect of the neuropeptide phoenixin and its receptor GPR173 during folliculogenesis. Reproduction.

[22] Ullah K (2017). Phoenixin-14 concentrations are increased in association with luteinizing hormone and nesfatin-1 concentrations in women with polycystic ovary syndrome. Clin. Chim. Acta.

[23] Kalamon N (2020). Levels of the neuropeptide phoenixin-14 and its receptor GRP173 in the hypothalamus, ovary and periovarian adipose tissue in rat model of polycystic ovary syndrome. Biochem. Biophys. Res. Commun..

[24] Kulinska KI (2021). Phoenixin as a new target in the development of strategies for endometriosis diagnosis and treatment. Biomedicines.

[25] Rybska M (2022). Canine cystic endometrial hyperplasia and pyometra may downregulate neuropeptide phoenixin and GPR173 receptor expression. Anim. Reprod. Sci..

[26] Wang M (2018). Phoenixin participated in regulation of food intake and growth in spotted scat, *Scatophagus argus*. Comp. Biochem. Physiol. Part B.

[27] Rajeswari JJ, Blanco AM, Unniappan S (2020). Phoenixin-20 suppresses food intake, modulates glucoregulatory enzymes, and enhances glycolysis in zebrafish. Am. J. Physiol. Reg. I.

[28] Rajaei S (2022). Mediatory role of the central NPY, melanocortine and corticotrophin systems on phoenixin-14 induced hyperphagia in neonatal chicken. Gen. Comp. Endocrinol..

[29] Wang M (2019). Phoenixin: Expression at different ovarian development stages and effects on genes related to reproduction in spotted scat, *Scatophagus argus*. Comp. Biochem. Physiol. Part B.

[30] Rajeswari JJ, Unniappan S (2020). Phoenixin-20 stimulates mRNAs encoding hypothalamo-pituitary-gonadal-hormones, is pro-vitellogenic, and promotes oocyte maturation in zebrafish. Sci. Rep..

[31] Breton TS (2021). Characterization of the G protein-coupled receptor family SREB across fish evolution. Sci. Rep..

[32] Watson CA, Hill JE, Graves JS, Wood AL, Kilgore KH (2009). Use of a novel induced spawning technique for the first reported captive spawning of *Tetraodon nigroviridis*. Mar. Genom..

[33] Sipos MJ (2019). Evaluation of cGnRH IIa for induction spawning of two ornamental *Synodontis* species. Aquaculture.

[34] Breton TS, DiMaggio MA, Sower SA, Berlinsky DL (2015). Brain aromatase (*cyp19a1b*) and gonadotropin releasing hormone (*gnrh2* and *gnrh3*) expression during reproductive development and sex change in black sea bass (*Centropristis striata*). Comp. Biochem. Physiol. Part A.

[35] Picha ME, Turano MJ, Tipsmark CK, Borski RJ (2008). Regulation of endocrine and paracrine sources of Igfs and Gh receptor during compensatory growth in hybrid striped bass (*Morone chrysops*× *Morone saxatilis*). J. Endocrinol..

[36] Breton TS (2019). Initiation of sex change and gonadal gene expression in black sea bass (*Centropristis striata*) exposed to exemestane, an aromatase inhibitor. Comp. Biochem. Physiol. Part A.

[37] Pfaffl MW (2001). A new mathematical model for relative quantification in real time RTPCR. Nucleic Acids Res..

[38] Hampton LMT, Finch MG, Martyniuk CJ, Venables BJ, Jeffries MKS (2021). Developmental thyroid disruption causes long-term impacts on immune cell functions and transcriptional responses to pathogen in a small fish model. Sci. Rep..

[39] Nouri M-Z, Kroll K, Webb M, Denslow N (2020). Quantification of steroid hormones in low volume plasma and tissue homogenates of fish using LC-MS/MS. Gen. Comp. Endocrinol..

[40] Ma H (2020). Phoenixin 14 inhibits ischemia/reperfusion-induced cytotoxicity in microglia. Arch. Biochem. Biophys..

[41] Sun G, Ren Q, Bai L, Zhang L (2020). Phoenixin-20 suppresses lipopolysaccharide-induced inflammation in dental pulp cells. Chem. Biol. Interact..

[42] Zhang B, Li J (2020). Phoenixin-14 protects human brain vascular endothelial cells against oxygen-glucose deprivation/reoxygenation (OGD/R)-induced inflammation and permeability. Arch. Biochem. Biophys..

[43] Yang F (2020). Phoenixin 14 inhibits high-fat diet-induced non-alcoholic fatty liver disease in experimental mice. Drug Des. Dev. Ther..

[44] Jais A, Brüning JC (2017). Hypothalamic inflammation in obesity and metabolic disease. J. Clin. Investig..

[45] Fu Y (2020). Gut hormone GIP induces inflammation and insulin resistance in the hypothalamus. Endocrinology.

[46] Wang, S., Liang, R. & Liu, H. Phoenixin-20 ameliorates brain infarction by promoting microglia M2 polarization in an ischemic stroke model. *Metab. Brain Dis.* (2022). 10.1007/s11011-022-00950-5 35334042

[47] Matsumoto M (2005). A conserved mRNA expression profile of SREB2 (GPR85) in adult human, monkey, and rat forebrain. Mol. Brain Res..

[48] Ceriani R, Calfún C, Whitlock KE (2021). *phoenixin*(*smim20*), a gene coding for a novel reproductive ligand, is expressed in the brain of adult zebrafish. Gene Expr. Patterns.

[49] Kubiczkova L, Sedlarikova L, Hajek R, Sevcikova S (2012). TGF-β—An excellent servant but a bad master. J. Transl. Med..

[50] Larco DO, Bauman BM, Cho-Clark M, Mani SK, Wu TJ (2018). GnRH-(1–5) inhibits TGF-β signaling to regulate the migration of immortalized gonadotropin-releasing hormone neurons. Front. Endocrinol..

[51] Ruan K, Bao S, Ouyang G (2009). The multifaceted role of periostin in tumorigenesis. Cell. Mol. Life Sci..

[52] Antonin W, Neumann H (2016). Chromosome condensation and decondensation during mitosis. Curr. Opin. Cell Biol..

[53] Zais DMW, Gause WC, Osborne LC, Artis D (2015). Emerging functions of amphiregulin in orchestrating immunity, inflammation, and tissue repair. Immunity.

[54] Ku GM, Pappalardo Z, Luo CC, German MS, McManus MT (2012). An siRNA screen in pancreatic beta cells reveals a role for gpr27 in insulin production. PLoS Genet..

[55] Chopra DG, Yiv N, Hennings TG, Zhang Y, Ku GM (2020). Deletion of Gpr27 in vivo reduces insulin mRNA but does not result in diabetes. Sci. Rep..

[56] Matsumoto M (2008). The evolutionarily conserved G protein-coupled receptor SREB2/GPR85 influences brain size, behavior, and vulnerability to schizophrenia. Proc. Natl. Acad. Sci..

[57] Chen Q (2012). SREB2/GPR85, a schizophrenia risk factor, negatively regulates hippocampal adult neurogenesis and neurogenesis dependent learning and memory. Eur. J. Neurosci..

[58] De La Fuente R, Eppig JJ (2001). Transcriptional activity of the mouse oocyte genome: Companion granulosa cells modulate transcription and chromatin remodeling. Dev. Biol..

[59] Breton TS, Berlinsky DL (2014). Characterizing ovarian gene expression during oocyte growth in Atlantic cod (*Gadus morhua*). Comp. Biochem. Physiol. Part D.

[60] Nagahama Y, Yamashita M (2008). Regulation of oocyte maturation in fish. Dev. Growth Differ..

[61] Clelland ES, Tan Q, Balofsky A, Lacivita R, Peng C (2007). Inhibition of premature oocyte maturation: A role for bone morphogenetic protein 15 in zebrafish ovarian follicles. Endocrinology.

[62] Peng Y, Ge W (2002). Epidermal growth factor and TGFα promote zebrafish oocyte maturation in vitro: Potential role of the ovarian activin regulatory system. Endocrinology.

[63] Mo X (2014). Leukemia inhibitory factor enhances bovine oocyte maturation and early embryo development. Mol. Reprod. Dev..

