# Supplementary material for: Phoenixin-14 alters transcriptome and steroid profiles in female green-spotted puffer (Dichotomyctere nigroviridis)
Source: Sci Rep. 2022 Jun 8;12:9454. doi: 10.1038/s41598-022-13695-z (PMC9177834; doi:10.1038/s41598-022-13695-z)
Supplement: Supplementary file 2 — Supplementary Information 2. [file 41598_2022_13695_MOESM2_ESM.pdf]

Supplementary Information for:

Phoenixin-14 alters transcriptome and steroid profiles in female green-spotted puffer (*Dichotomyctere nigroviridis*)

Timothy S. Breton[1*], Casey A. Murray[2], Sierra R. Huff[1], Anyssa M. Phaneuf[1], Bethany M. Tripp[1], Sarah J. Patuel[3], Christopher J. Martyniuk[3], Matthew A. DiMaggio[2]

[1]Division of Natural Sciences, University of Maine at Farmington, Farmington, ME, 04938, USA

[2]Tropical Aquaculture Laboratory, Program in Fisheries and Aquatic Sciences, School of Forest Resources and Conservation, Institute of Food and Agricultural Sciences, University of Florida, Ruskin, FL, 33570, USA

[3]Center for Environmental and Human Toxicology, Department of Physiological Sciences, College of Veterinary Medicine, University of Florida, Gainesville, FL, 32611, USA

*Corresponding author: timothy.breton@maine.edu

Table S1. Standard RNA-sequencing quality control parameters for all hypothalamus control (HC), hypothalamus PNX-14 treatment (HT), ovary control (OC), and ovary PNX-14 treatment (OT) samples.

Sample name	Raw reads	Clean reads	Raw bases	Clean bases	Error rate (%)	Q20(%)	Q30(%)	GC content(%)
HT6	23683579	22970487	7.1	6.9	0.03	97.46	93.34	51.19
HT5	20233591	19809451	6.1	5.9	0.03	97.5	93.48	52.4
HT4	22458378	21959389	6.7	6.6	0.03	97.11	92.61	52.36
HT3	21748233	21262948	6.5	6.4	0.03	97.41	93.27	52.67
HT2	21599044	21152493	6.5	6.3	0.03	97.19	92.57	52.57
HT1	20684564	20207854	6.2	6.1	0.03	96.93	92.31	52.07
OT2	20340845	20118149	6.1	6	0.03	97.92	94.05	52.91
OT3	21352473	20717037	6.4	6.2	0.03	97.83	93.99	54.69
OT4	22998277	22373548	6.9	6.7	0.03	97.58	93.59	53.62
OT5	26038156	25522447	7.8	7.7	0.03	97.74	93.72	54.45
OT6	22307208	21874228	6.7	6.6	0.03	97.77	93.79	54.07
HC2	21976376	21080348	6.6	6.3	0.03	97.01	92.36	50.57
HC3	25224090	24257007	7.6	7.3	0.03	97.05	92.54	51.22
HC1	21877547	21110065	6.6	6.3	0.03	97.63	93.75	51.31
HC6	26396557	25784808	7.9	7.7	0.03	97.4	93.2	52.36
HC4	23231680	22684948	7	6.8	0.03	97.57	93.52	51.24
HC5	26491332	25970758	7.9	7.8	0.03	97.49	93.43	52.6
OC1	22696692	21968313	6.8	6.6	0.03	97.46	93.31	52.91
OC3	22517492	21841644	6.8	6.6	0.03	97.41	93.16	53.23
OC2	21380722	20824646	6.4	6.2	0.03	97.8	94.03	53.93
OC5	20824342	20040480	6.2	6	0.03	97.27	92.88	53.37
OC6	27026145	26482016	8.1	7.9	0.03	97.78	93.98	54.05

Table S2. Gene names, primer sequences, PCR product sizes (bp), mean cycle threshold values (Ct), and PCR efficiencies (eff. %) for all hypothalamus, ovary, and liver qPCR assays. Mean Ct refer to the 1/40 diluted standard curve value for each assay.

			Mean Ct (eff. %)		
Gene	Sequence (5'-3')	bp	Hypo	Ovary	Liver
acta1	F - CGTACCACAGGGATTGTGCT	129		22.62 (105.7)	
	R - CAGGTAGTCGGTCAGATCGC				
dkk3a	F - TGAGGAGACGGAGAGCATCA	150	26.93 (85.0)		
	R - GCCTCTCCTCTGGTTGCATT				
dpydb	F - AGAGCGGTTCCAGAGGAGAT	118	29.59 (93.7)	29.60 (106.8)	
	R - GACAGAACTGTAACCCGGCA				
eef1a	F - TGAGGCCGGTATCTCCAAGA	132	16.98 (90.0)	19.87 (93.9)	15.24 (91.6)
	R - AAACGTGCCTGACTGTAGGG				
esr1	F - GGAGGTCCATCCACTACCCT	144			21.88 (99.9)
	R - TTAGCTGGAGCATGCGGAAA				
esr2a	F - CTGTCCTGCGACCAATCAGT	87			27.50 (90.5)
	R - CCGACTTCGTAGCATTTGCG				
esr2b	F - TGGTCTACAACCTGCTGCTG	81			25.87 (92.4)
	R - GGAACTGGTCGGAGTTGGAG				
isr2a	F - GAAGCTGATCGAGGAGGAGC	107		20.82 (100.7)	
	R - AAACCTCCCTGAACAAGGGC				
safb	F - CCGCTATGGACGCTCAGATT	143		25.64 (102.0)	
	R - AGTGCTGACCCTCTCTGTCT				
shbg	F - CAGGTATCGGGGGATTCAGC	142			17.59 (89.9)
	R - TCACACCTTCGGGGTCAAAG				
slc26a2	F - TCCCTGTCGGAGATGTTTGC	148		26.64 (100.1)	
	R - ACTCCTTGACCAGCGTCTTG				
slc31a1	F - TAAAACTGTTGGGCAGCGGA	135		25.73 (103.1)	
	R - GCCACGGCGATACACAGATA				
srl	F - GGAGATGTCGCTGATGGAGG	85		25.83 (91.6)	
	R - GCCGTGCTGCCTTATAAACG				
tgfb3	F - GCTCTACCAGATCGTGAGGC	148	26.50 (103.0)		
	R - ATTGCTGCCTCGGTTCATCA				
vtgab	F - GGGGCAAGAACTGCAAACAG	97			16.35 (91.1)
	R - TTGCCCCAGCTCATCTTCAG				
vtgc	F - TACTGGAGGCTGAGGAGGAC	89			18.69 (94.6)
	R - AAAGCCGTAGACGTTGCTGA				

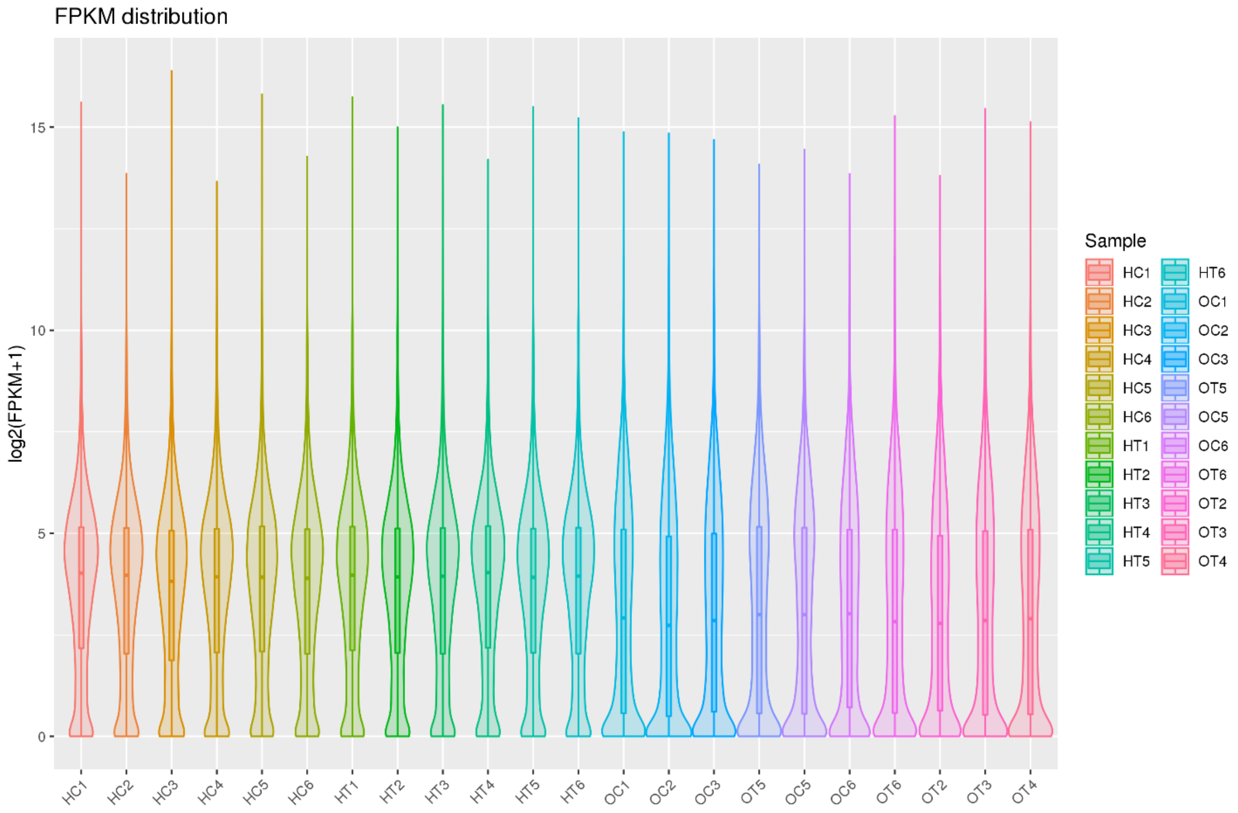

Fig. S1. Distribution of log2 Fragments Per Kilobase of transcript per Million mapped reads for all hypothalamus control (HC), hypothalamus PNX-14 treatment (HT), ovary control (OC), and ovary PNX-14 treatment (OT) samples.

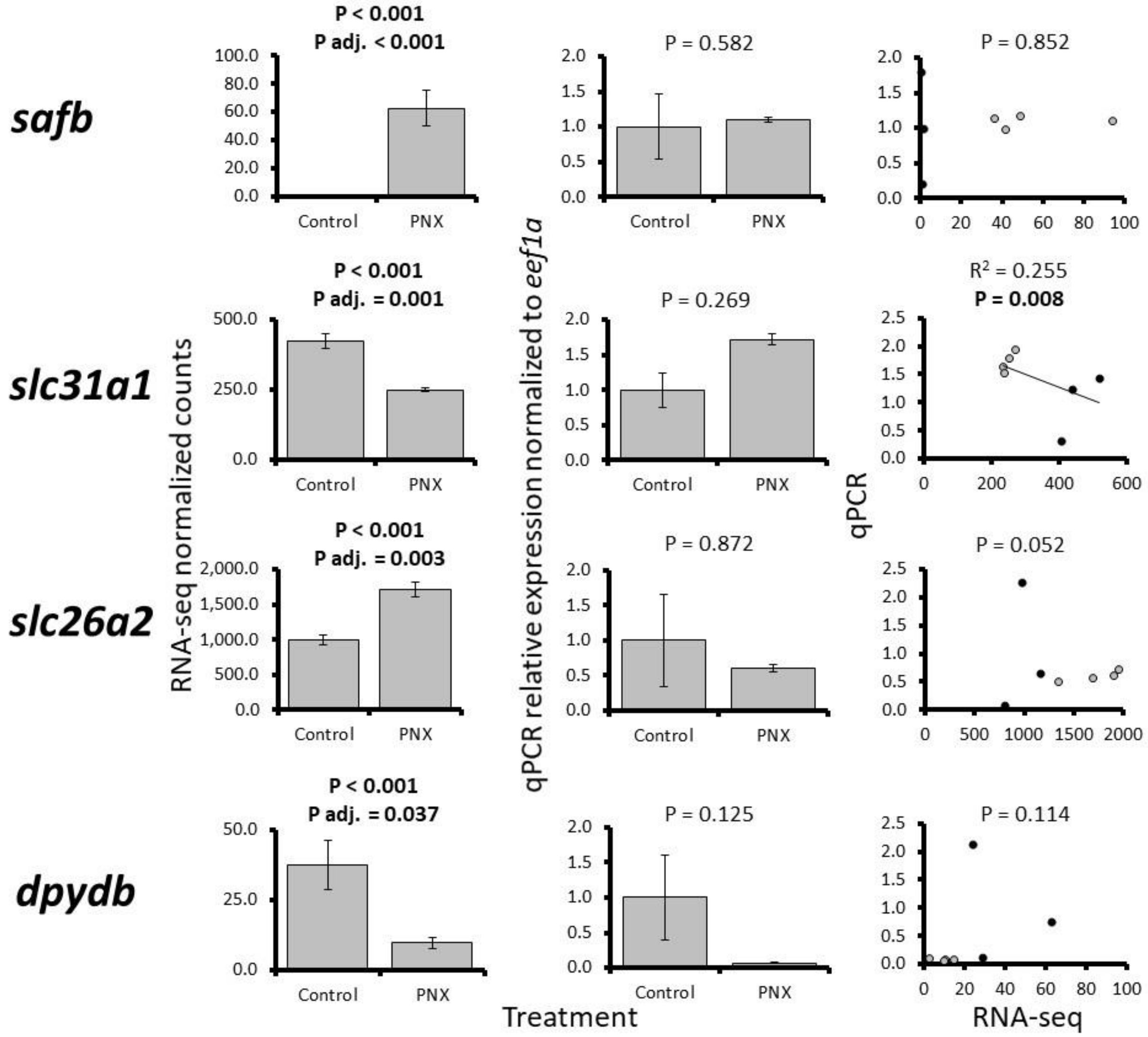

Fig. S2. RNA-seq normalized count data (first column), relative mRNA expression in qPCR (second column), and linear regression analyses (third column) of four ovary genes (safb, slc31a1, slc26a2, and dpydb) that were first identified as differentially expressed by RNA-seq but could not be confirmed through qPCR. Each bar represents the mean ± standard error and significance was assessed at $P < 0.05$. P adjusted (adj.) refers to the multiple test correction in RNA-seq differential expression analyses. For regression analyses, black and gray circles refer to control and PNX-14 treated samples, respectively.

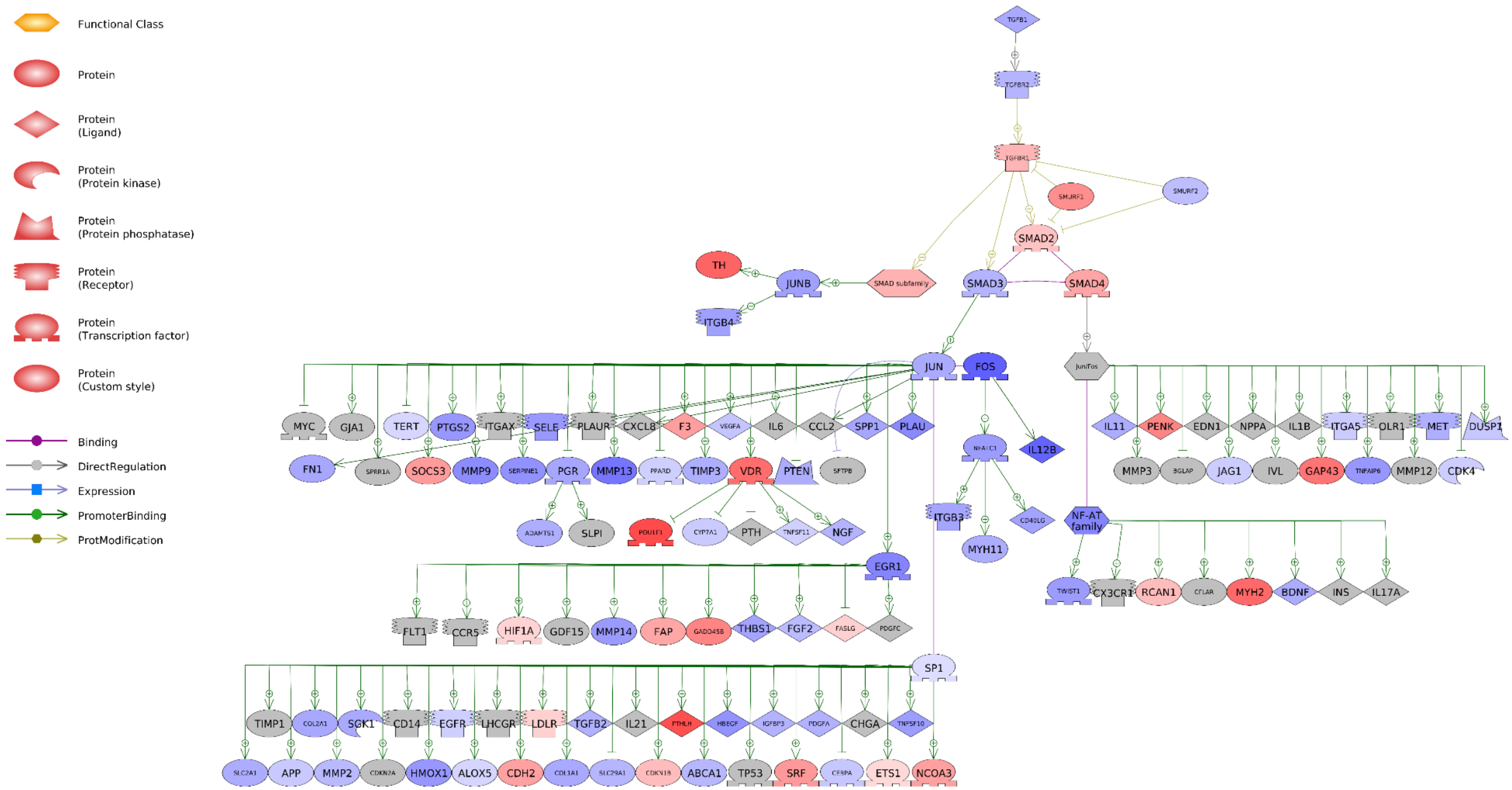

Fig. S3. TGFB1-TGFBR1/AP-1 expression targets pathway identified in the ovary. Blue indicates a significantly downregulated gene in the transcriptome (darker shade indicate greater change compared to control), while red indicates an upregulated gene. Shapes and arrows are identified in the legend. This is a proposed mechanism based only on transcriptomic data.

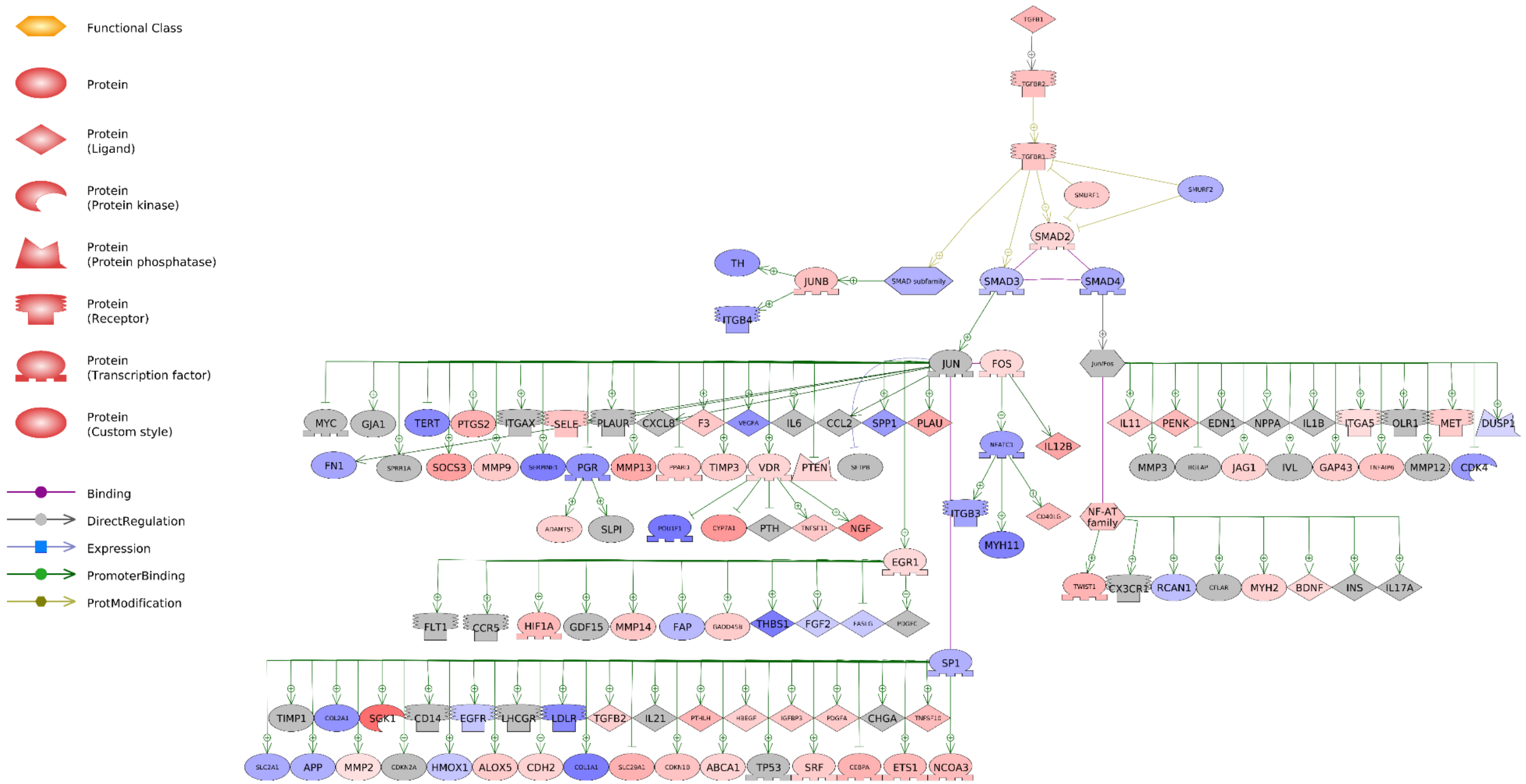

Fig. S4. TGFB1-TGFBR1/AP-1 expression targets pathway identified in the hypothalamus. Blue indicates a significantly downregulated gene in the transcriptome (darker shade indicate greater change compared to control), while red indicates an upregulated gene. Shapes and arrows are identified in the legend. This is a proposed mechanism based only on transcriptomic data.

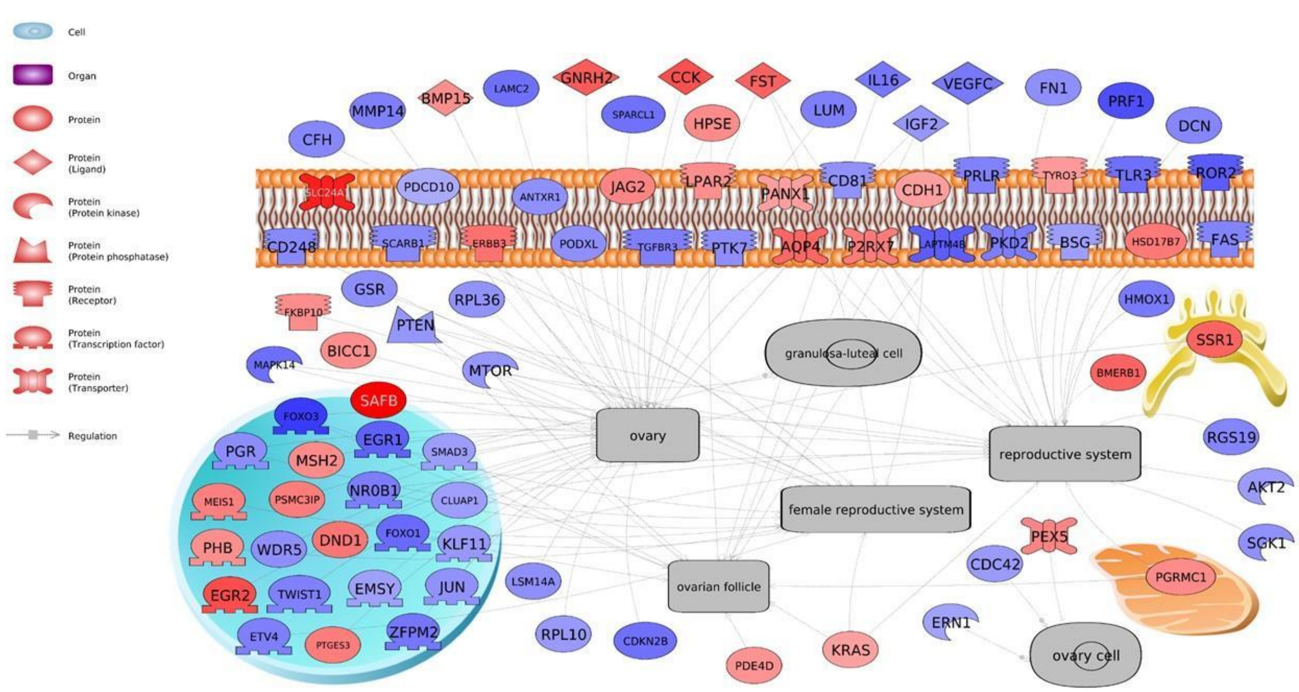

Fig. S5. Networks associated with reproduction in the ovary following PNX treatment. Red indicates an upregulated gene in the network (darker shade indicates greater log2 fold change compared to control), while blue indicates a downregulated gene. Shapes and arrows are identified in the legend to the left. This is a proposed mechanism based only on transcriptomic data.

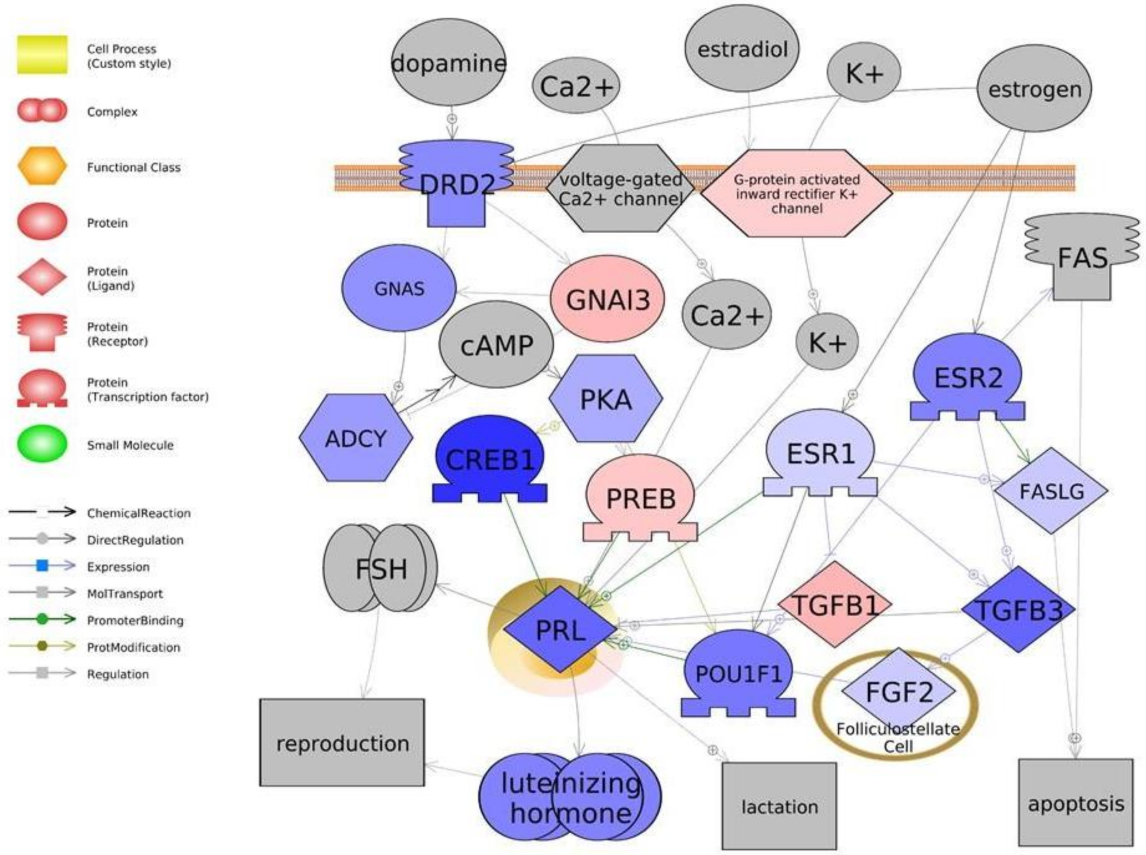

Fig. S6. Networks associated with reproduction in the hypothalamus following PNX treatment. Red indicates an upregulated gene in the network (darker shade indicates greater log2 fold change compared to control), while blue indicates a downregulated gene. Shapes and arrows are identified in the legend to the left. This is a proposed mechanism based only on transcriptomic data.
